# Isolation and Identification of *Cis-*2,5-Diketopiperazine from a Novel *Bacillus* Strain and Synthesis of Its Four Stereoisomers

**DOI:** 10.3390/md23060234

**Published:** 2025-05-29

**Authors:** Alan M. C. Obled, Refaat B. Hamed, Edward Spence, Marija K. Zacharova, Sunil V. Sharma, Yunpeng Wang, Rosemary Lynch, Helen Connaris, Adina Tatheer, Marie-Lise Bourguet-Kondracki, Gordon J. Florence, Rebecca J. M. Goss

**Affiliations:** 1 School of Chemistry & BSRC, and Integrated Institute of Engineering, University of St Andrews, North Haugh, St Andrews, Fife KY16 9ST, UK; 2 Faculty of Life Sciences, University of Bradford, Bradford, West Yorkshire BD7 1DP, UK; 3 Department of Chemical and Environmental Engineering, University of Nottingham, Nottingham NG7 2RD, UK; 4 Engineering and Physical Sciences Research Council (EPSRC), UKRI, Swindon SN2 1FL, UK; 5 Laboratoire Molécules de Communication et Adaptation des Micro-Organismes, UMR 7245 CNRS, Muséum National d’Histoire Naturelle, 57 Rue Cuvier (C.P. 54), 75005 Paris, France

**Keywords:** *Bacillus*, marine sponge bacteria, diketopiperazine, cyclopeptide, antimicrobial

## Abstract

The *Bacillus horneckiae*-like strain 2011SOCCUF3 was isolated from the marine sponge *Spongia officinalis* and its metabolome was studied for secondary metabolites with antimicrobial activity. Culturing in the presence of Diaion HP-20 resin and purifying the culture extract identified cyclo-phenylalanine-proline (cyclo-(Phe-Pro)), a 2,5-diketopiperazine (2,5-DKP), isolated as a major metabolite. Further, LCMS analysis of the extract showed the presence of two isomers of the molecule in the culture broth. To confirm the stereochemistry of the isomers observed in the natural extract, all four stereoisomers of cyclo-(Phe-Pro) were synthesised. NMR and LCMS studies identified the presence of both *cis-* and *trans*-cyclo-(Phe-Pro) isomers. Stability and epimerisation studies on synthetic isomers and the effect of culturing conditions suggested that the less stable *cis* isomer was naturally produced, which epimerised in culture broth.

## 1. Introduction

Cyclic dipeptides (or 2,5-diketopiperazines or 2,5-DKPs) are a large class of natural products associated with several biological properties [1], including antibiotic [2,3], antifungal [4,5,6], antiviral [7,8], anticancer [9,10,11], and herbicidal [12] activities. These smallest cyclic peptides are produced by bacteria, fungi, plants, and animals [1]. As well as representing intrinsically bioactive natural products, 2,5-DKPs provide useful fragments for molecular drug design for a wide variety of biomedical applications [13,14]. Their antimicrobial activity has been of particular interest, with antimicrobial resistance being predicted to become a greater burden on public health than cancer by 2050 [15]. Furthermore, multiple 2,5-DKPs have been found to be naturally occurring antifouling agents, with both natural and synthetic analogues being considered as promising greener antifouling solutions for marine industries [16,17,18].

Cyclo-l-phenylalanine-l-proline (cyclo-(l-Phe-l-Pro)) is a 2,5-DKP isolated from various bacteria including *Streptomyces* [19], *Vibrio* [20], *Bacillus* [21], *Nocardiopsis* [22], as well as other microorganisms including fungi [16]. Apart from antifouling properties [16,17,18], it has also been found to play a role in quorum sensing in *Vibrio* spp. [20,23,24]. Additionally, it has antifungal [4,8] and antibacterial properties, including activity against MRSA [19].

A few biosynthetic pathways of 2,5-DKP elucidated so far include both non-enzymatic and enzymatic pathways. Non-enzymatic pathways involve spontaneous cyclisation of linear dipeptide precursors. In enzyme-catalysed pathways, the final cyclisation is catalysed by non-ribosomal peptide synthetases (NRPSs) or cyclic dipeptide synthases (CDPSs) [13], with the latter using aminoacyl-tRNAs as substrates for 2,5-DKP assembly [22]. Remarkably, while 2,5-DKPs, particularly those containing proline, are prone to epimerisation, the vast majority of bioactive 2,5-DKPs isolated from natural sources are reported to have the more strained *cis* (l,l) stereochemistry, and in the rare example of both isomers being isolated, the *cis* isomer was the major one [21,25].

This study describes the isolation and characterisation of the *cis-*cyclo-(l-Phe-l-Pro) isomer from the novel *Bacillus* strain. The isolated natural compound was compared to four synthetic stereoisomers. Under mildly basic conditions, isomerisation of the synthetic standards was observed over time, with a time course being determined. This observed isomerisation may explain why both *cis* and *trans* isomers are observed in the culture extract.

## 2. Results

### 2.1. Isolation and Identification of Cyclo-(Phe-Pro)

The *Bacillus horneckiae*-like strain 2011SOCCUF3, isolated from the marine sponge *Spongia officinalis* [26], was grown in a TCOATSS medium in the presence of Diaion HP-20 resin (5% *w*/*v*). The methanolic extracts of the cell pellet and resin were used for bioactivity-guided (antibacterial) fractionation, gel filtration, and reverse phase purification. Upon LCMS analysis, a predominantly single species with a mass of *m*/*z* 245.1279 was identified as a major constituent. The MS/MS fragmentation pattern associated with this mass showed two predominant species consistent with [M-CO+H]^+^ and [M-CO-proline+H]^+^ fragments (*m*/*z* 217.08 and 120.04, respectively). ^1^H NMR indicated the presence of an aromatic amino acid, pointing to phenylalanine as the second amino acid, which was consistent with the observed masses. ^1^H and ^1^H-^1^H COSY NMR indicated that the compound was cyclo-(Phe-Pro).

### 2.2. Chemical Synthesis of Cyclo-(Phe-Pro) Isomers

**Scheme 1:**
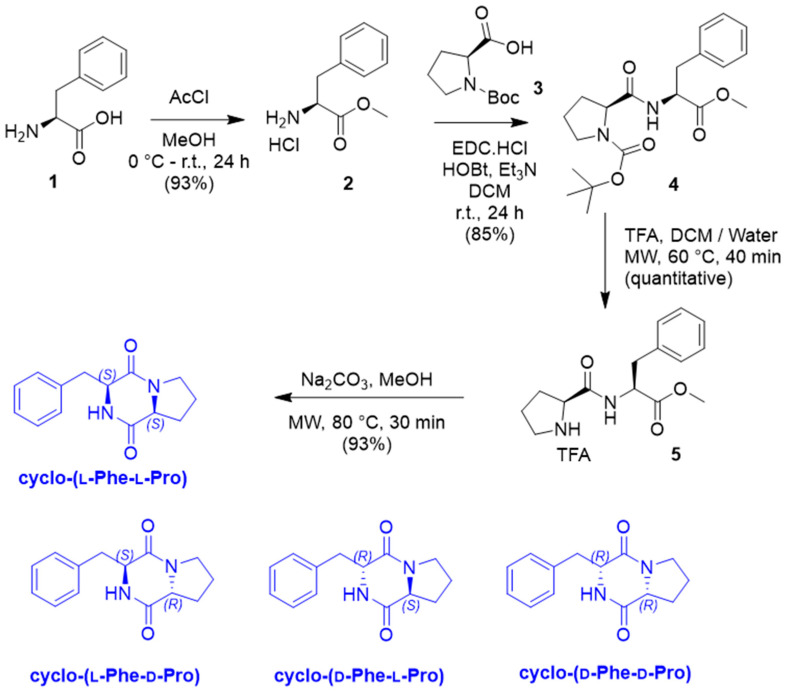
General representation for the synthesis of cyclo-(Phe-Pro) from unprotected phenylalanine, the four different stereoisomers synthesised separately are shown in blue.

To assign the stereochemistry of the isolated 2,5-DKP isomer, a synthesis of all four stereoisomers was carried out. The four-step synthesis involved methyl esterification of phenylalanine (**1**), peptide coupling to a Boc-protected proline, Boc deprotection, and final cyclisation (Scheme 1). The reaction products were monitored by ^1^H NMR and LCMS, with close attention paid to product stereochemistry. After the first amide coupling, two sets of NMR peaks corresponding to rotamers of Boc-Pro-Phe-OMe (**4**) were observed. The presence of rotamers due to the Boc-protected proline that restricted rotation along the amide bond has been previously reported [27] and was further confirmed by the two sets of signals (**4**) collapsing to one (**5**) after Boc deprotection. During the final cyclisation step, epimerisation of the 2,5-DKP was observed as all four syntheses resulted in mixtures of isomers, with *trans* (l,d or d,l) being the major isomer (in ~4–5: 1 ratio). Gratifyingly, *cis* and *trans* isomers were separable by reverse phase HPLC due to their difference in polarity [28]. Purification by reverse phase chromatography (semi-preparative HPLC) allowed for effective separation of *cis* and *trans* isomers and access to target products. Use of a phenyl-hexyl column (XBridge Prep Phenyl-hexyl 5 µm, 250 × 10.0 mm) with a 50 min gradient from 5% to 60% methanol in water provided optimum separation of the two isomers eluting at 36 min and 39 min. Detailed NMR analysis confirmed that two peaks were *trans* and *cis* isomers, respectively.

### 2.3. Establishing the Stereochemistry of Isolated Natural Cyclo-(Phe-Pro)

Comparison of the ^1^H NMR spectrum of the isolation product to that of the synthetic standards confirmed that the natural product was the *cis-*cyclo-(l-Phe-l-Pro) isomer (Table 1). The ^1^H NMR chemical shifts of CH_α_ (H_6_ and H_9_) on the proline and phenylalanine residues were used to distinguish between *trans* and *cis* isomers. In particular, CH_α_ (H_6_) on the proline moiety showed distinct chemical shifts of 2.60 ppm for the *trans* isomer and 4.07 ppm for the *cis* isomer. This difference in the chemical shifts for the *α*-proton comes from the 3D configuration of the molecule; the conformational preference of the *cis* and *trans* isomers is substantially different [29]. Unlike the extended *cis* configuration, the phenyl ring stacks on the top of the 2,5-DKP ring in the *trans* configuration [30,31]. The aromaticity is responsible for an electronic effect known as the aromatic ring current (or anisotropy) that shields protons that are closer to the inside of an aromatic ring. These results are consistent with previous literature [21,23,32].

To probe if the isolated *cis-*2,5-DKP product was the only or major isomer produced by the strain, a co-elution study was performed by LCMS using both culture broth and a methanolic extract of *Bacillus* cultures with synthetic samples. During these experiments, to our surprise, both the culture broth and the methanolic extract indicated that the *trans-*cyclo-(l-Phe-d-Pro) isomer was predominant and that the *cis-*cyclo-(l-Phe-l-Pro) isomer was present in only a minor amount, with a ratio of approximately 80–100:1. Isolation of the *cis-*cyclo-(l-Phe-l-Pro) isomer was confirmed by NMR.

**Figure 1:**
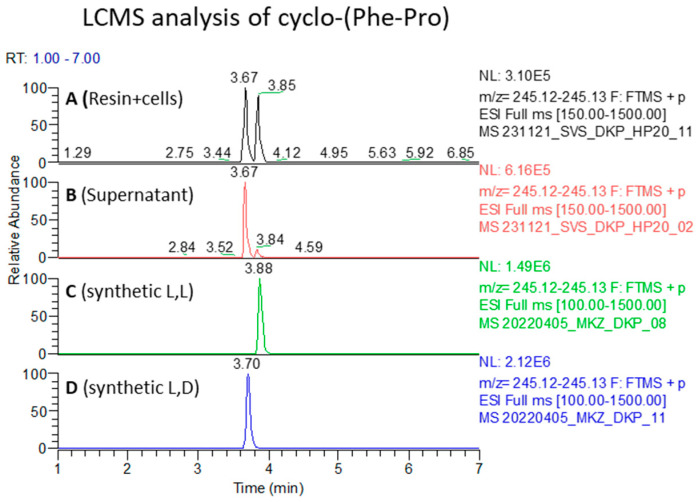
Comparison of the LCMS retention times of cyclo-(Phe-Pro). EIC traces of the methanolic extract of the Diaion HP-20 resin and the cell pellets (trace **A**), culture supernatant (trace **B**), synthetic *cis* (l,l) (trace **C**), and synthetic *trans* (l,d) isomer (trace **D**). Mass range displayed *m*/*z* = 245.12–245.13. l,d isomer elutes at a retention time of 3.62–3.84 min. l,l retention time is 3.84–3.95 min.

Intrigued by these results, we further probed the effect of culturing conditions and resin treatments on the production of the target natural product. To this end, Diaion HP-20 resin was used untreated, sterilised by autoclave with media, or sterilised by UV exposure (for 2 h). All cultures were grown in the presence of 5% *w*/*v* resin. Cell pellets and resin were collected by centrifugation and corresponding methanolic extracts were analysed by LCMS (Figure 1). The results indicated that significantly higher amounts (~50%) of *cis-*cyclo-(l-Phe-l-Pro) were present in the samples where UV-treated resin was used. All other cultures contained the *cis* isomer only as a minor component (5–10%), with the major isomer being the *trans* isomer.

Diaion HP-20 resin is commonly used as an effective adsorbent for hydrophobic molecules and natural products. Use of hydrophobic resins as an adsorbent is known to influence microbial growth and secondary metabolite production [33]. We postulated that cyclo-(Phe-Pro) is released in the extracellular media by passive diffusion [34]. Adsorption on the polystyrene–divinylbenzene matrix of the Diaion HP-20 resin protects the stereochemical integrity of the molecules. In the absence of the resin, cyclo-(Phe-Pro) remains prone to epimerisation in solution. We further investigated the stability and epimerisation of cyclo-(Phe-Pro) using the synthetic *cis* and *trans* isomers.

### 2.4. Probing the Epimerisation of Cyclo-(Phe-Pro)

During the synthesis, noticeable epimerisation was observed for the final cyclisation step. Attempts to optimise the final cyclisation of cyclo-(l-Phe-l-Pro) were made in order to minimise the epimerisation. When performed using microwave instead of conventional heating [35], faster cyclisation was achieved in methanol in the presence of solid sodium carbonate. Reaction temperature not only affected the rate of cyclisation, but also the extent of epimerisation. At 80 °C (or higher), the ratio of *cis* to *trans* was ~1:3 after 30 min with >90% conversions. Reducing the temperature to 60 °C made it possible to detect more of the kinetic product as the ratio of *cis* to *trans* was around 2:1 in favour of the *cis* product (based on LCMS), albeit only 38% conversion was observed. Extending the reaction times to improve the conversion led to increased epimerisation. Hence, cyclisation reactions were carried out at 80 °C.

**Figure 2:**
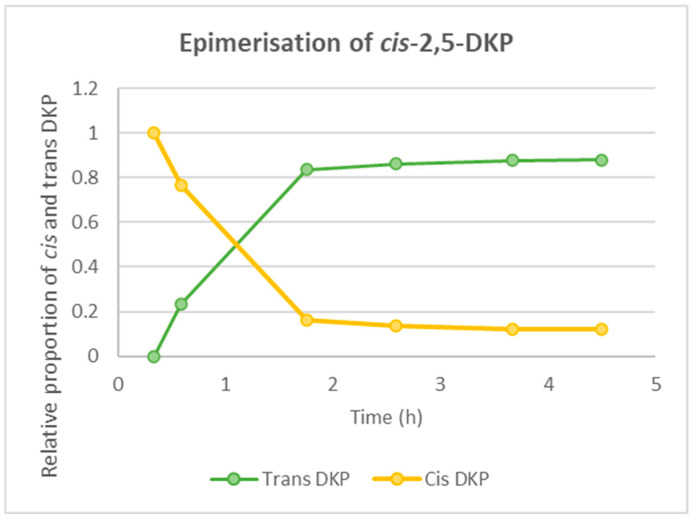
Epimerisation of 2,5-DKP under conventional heating (Na_2_CO_3_ in MeOH, 70 °C)**.** The ratio of *cis* (yellow) and *trans* (green) isomers were determined using the relative peak area generated by LCMS analysis.

Fortunately, epimerisation products proved to be separable by reverse phase HPLC using a C18 column. Under mild basic conditions (Na_2_CO_3_ in MeOH), purified *cis-*cyclo-(l-Phe-l-Pro) epimerised into the corresponding *trans* isomer (>85% within 2 h) (Figure 2). Epimerisation was not detected under acidic and neutral conditions (monitored up to 4 days at ambient temperature).

**Figure 3:**
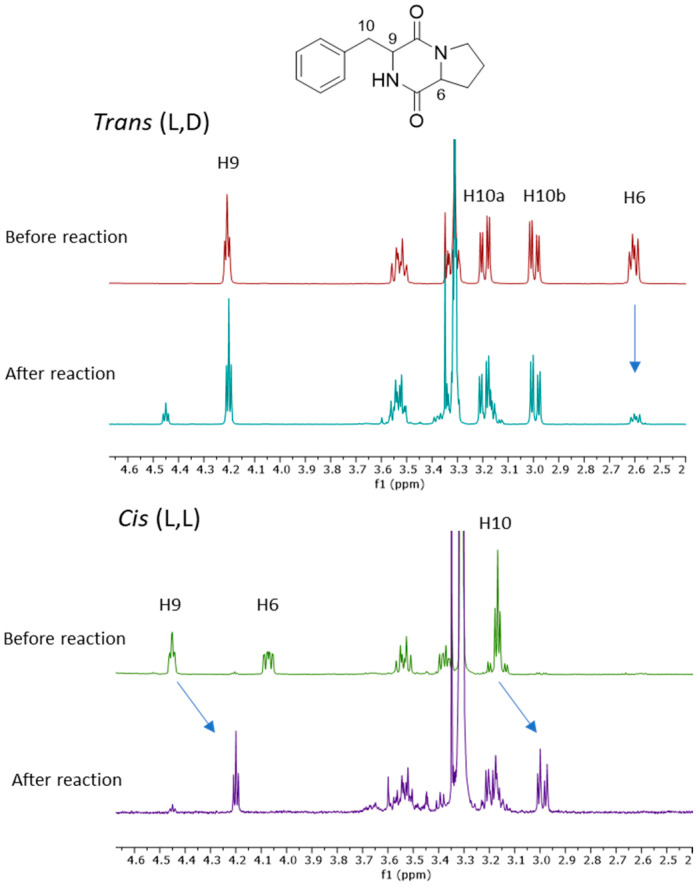
Studies of deuteration of 2,5-DKP. The proline Cα proton (H6) undergoes faster deuterium exchange, while *cis-*2,5-DKP readily epimerises into the *trans* isomer. Arrows indicate proton shift.

To assess product stabilities and the relative rates of different epimerisation reactions, the purified synthetic 2,5-DKPs were used in deuteration reactions. Gentle heating under basic conditions (solid Na_2_CO_3_ in MeOD-*d*_4_) resulted in fast deuteration of the proline Cα proton (H_6_), while the phenylalanine Cα proton (H_9_) epimerised more slowly. These results are consistent with literature indicating that proline-containing 2,5-DKPs were particularly prone to epimerisation [21] (Figure 3). Comparison of the ^1^H NMR of the reaction mixture indicated that >90% of the *cis-*2,5-DKP isomer epimersied into the *trans* isomer, along with the deuterium exchange of H_6_. Reaction of the *trans*-2,5-DKP isomer resulted in a high degree of H to D exchange at the proline Cα proton (H_6_); however, epimerisation was not prominent. These results further confirm that proline α-H_6_ is more prone to deuteration compared to α-H_9_, while the *trans* isomer is more stable [31,36] compared to the *cis* isomer.

## 3. Discussion

The novel *Bacillus* strain was found to produce 2,5-DKP cyclo-(Phe-Pro) that uniquely contained two isomers: the less common cyclo-(l-Phe-d-Pro) isomer along with the cyclo-(l-Phe-l-Pro) isomer. This study provides insight into the production of the *cis-*2,5-DKP isomer that rapidly epimerises in culture media. These data are supported by extensive synthetic work covering the synthesis of all four stereoisomers of 2,5-DKP for comparison with the two naturally occurring isomers. There might be multiple explanations for this unusually observed pattern that stem from both the stabilities of the isomers shown in this work and the limited knowledge about the biological functions and biosynthesis of 2,5-DKPs. One explanation could be that the final cyclisation of 2,5-DKPs can proceed spontaneously as well as enzymatically. This, combined with the susceptibility of proline-containing 2,5-DKPs to epimerisation, may skew the ratio of isomers towards the more stable *trans* isomer. Another possible argument might relate to the potential function of this natural product as an antifoulant. One might expect an isomer with high antimicrobial and antifouling activity to be produced at elevated concentrations. This would be particularly important to the bacterium’s symbiotic relationship with the sponge host in its natural environment, where high sea water throughput results in the demand for high levels of natural product production since it is being continuously diluted as the sponge filters water. Under these circumstances, the (likely enzymatic) production of the most active isomer might be favoured. The culturing conditions selected in this study are not representative of the marine environment; however, toxicity may be overcome by downregulating the production and epimerisation of a compound to its potentially less toxic isomer.

## 4. Materials and Methods

### 4.1. Strain Isolation and Culturing

The *Bacillus horneckiae*-like strain 2011SOCCUF3 was isolated from the marine sponge *Spongia officinalis*. The sponge was collected in October 2011 on the French coast near Marseille (Calanque de Cortiou) while searching for heavy metal-tolerant bacteria. 2011SOCCUF3 was reported to be tolerant to copper [26].

The strain was routinely maintained on marine agar (peptone 5 g/L, yeast extract 1 g/L, Instant Ocean^®^ sea salt 33 g/L, agar 15 g/L). TCOATSS was used as the medium for culturing (tryptone 4 g/L, casein 4 g/L, oatmeal 10 g/L, soluble starch 10 g/L, and trace element solution 1 mL/L; trace element solution: ZnCl_2_ 40 mg/L, FeCl_2_·4H_2_O 170 mg/L, CuCl_2_·2H_2_O 10 mg/L, Na_2_B_4_O_7_·10H_2_O 10 mg/L, MnCl_2_·4H_2_O 10 mg/L, (NH_4_)_6_Mo_7_O_24_·4H_2_O 10 mg/L).

### 4.2. Fractionation and Isolation of 2,5-DKP

The cultures were grown for 4 days (28 °C, 200 rpm) in 2 L non-baffled flasks (total volume 10 L) with Diaion HP-20 resin added at 50 g/L. The cultures were centrifuged and the cell pellets and resins were resuspended in methanol. The methanol extracts were dried *in vacuo* and resuspended in a minimum volume of water. The aqueous solution was loaded on a Diaion HP-20 column (300 g resin equilibrated with water) and eluted with a methanol–water gradient (2 L portions of 25%, 50%, 75%, and 100% methanol). A total of 22 fractions were collected (~400 mL each). The collected fractions (20 mL from each fraction) were dried *in vacuo* and the remaining residue was resuspended in 100–200 μL of 50% methanol–water. A total of 15 μL of each fraction was screened using disc diffusion assays. Active fractions were combined and further fractionated with a Sephadex LH-20 resin column using methanol as an eluant. A major product was isolated after the gel filtration purification. It was identified as cyclo-(Phe-Pro) by LCMS and NMR analysis.

### 4.3. General Procedure for the Cyclisation of Pro-Phe-OMe to Cyclo-(Phe-Pro)

The synthesis of the linear dipeptide Pro-Phe-OMe·TFA is described below. In a glass microwave vial containing a stirrer bar, solid Na_2_CO_3_ (5 eq.) was added to a solution of Pro-Phe-OMe·TFA (1 eq.) in MeOH (~6 mL/mmol). The vial was sealed with an aluminium crimp cap and the reaction was heated at 80 °C for 30 min in a Biotage microwave reactor. The solvent was removed *in vacuo*. The white solid was dissolved in 10 mL of water and extracted with DCM (5 times). The organic phases were combined and dried over MgSO_4_ before being evaporated *in vacuo*. A white solid containing 2,5-DKP was obtained. Purification by reverse phase semi-preparative HPLC (XBridge Phenyl-hexyl column, 5 µm, 250 × 10.0 mm), with a 50 min gradient from 5% to 60% methanol in water, was used for the isolation of pure diastereomers.

Cyclo-(l-Phe-l-Pro): HPLC elution time was 39 min. **^1^H NMR (500 MHz, MeOD-*d*_4_)** δ 7.31–7.19 (m, 5H, ArH), 4.45 (t, *J* = 4.3 Hz, 1H, H_9_), 4.07 (ddd, *J* = 10.7, 6.3, 1.6 Hz, 1H, H_6_), 3.54 (dt, *J* = 12.0, 8.4 Hz, 1H, H_3A_), 3.40–3.33 (m, 1H, H_3B_), 3.17 (t, *J* = 4.9 Hz, 2H, H_10_), 2.13–2.06 (m, 1H, H_5A_), 1.84–1.76 (m, 2H, H_4_), 1.26–1.14 (m, 1H, H_5B_) ppm; **^13^C NMR (126 MHz, MeOD-*d*_4_)** δ 170.9 (C=O), 166.9 (C=O), 137.3 (C*_arom_*), 131.1 (CH*_arom_*), 129.4 (CH*_arom_*), 128.1 (CH*_arom_*), 60.1 (CH), 57.7 (CH), 46.0 (CH_2_), 38.2 (CH_2_), 29.4 (CH_2_), 22.8 (CH_2_) ppm; **HRMS (ESI+)** *m*/*z* calculated for C_14_H_17_N_2_O_2_ [M+H]^+^ 245.1285 was found to be 245.1283. Literature pertaining to NMR is available for CDCl_3_ [21] or MeOD-*d*_4_ [32].

Cyclo-(l-Phe-d-Pro): HPLC elution time was 36 min. **^1^H NMR (500 MHz, MeOD-*d*_4_)** δ 7.34–7.27 (m, 3H, ArH), 7.21–7.16 (m, 2H, ArH), 4.21 (t, *J* = 4.7 Hz, 1H, H_9_), 3.53 (dt, *J* = 11.6, 8.6 Hz, 1H, H_3A_), 3.32 (m, 1H, H_3B_), 3.19 (dd, *J* = 13.7, 4.8 Hz, 1H, H_10A_), 3.00 (dd, *J* = 13.7, 4.7 Hz, 1H, H_10B_), 2.60 (dd, *J* = 10.6, 6.3 Hz, 1H, H_6_), 2.00–1.95 (m, 1H, H_5A_), 1.92–1.85 (m, 1H, H_4A_), 1.72–1.57 (m, 2H, H_4B,_ H_5B_) ppm; **^13^C NMR (126 MHz, MeOD-*d*_4_)** δ 171.3 (C=O), 167.4 (C=O), 136.7 (C*_arom_*), 131.3 (CH*_arom_*), 129.6 (CH*_arom_*), 128.5 (CH*_arom_*), 59.7 (CH), 59.1 (CH), 46.1 (CH_2_), 41.0 (CH_2_), 29.8 (CH_2_), 22.5 (CH_2_) ppm; **HRMS (ESI+)** *m*/*z* calculated for C_14_H_17_N_2_O_2_ [M+H]^+^ 245.1285 was found to be 245.1279. Literature pertaining to NMR was reported for CDCl_3_ [21].

Cyclo-(d-Phe-d-Pro): HPLC elution time was 39 min. **^1^H NMR (500 MHz, MeOD-*d*_4_)** δ 7.30–7.20 (m, 5H, ArH), 4.45 (td, *J* = 5.1, 2.1 Hz, 1H, H_9_), 4.07 (ddd, *J* = 10.9, 6.4, 2.0 Hz, 1H, H_6_), 3.54 (dt, *J* = 11.9, 8.4 Hz, 1H, H_3A_), 3.41–3.33 (m, 1H, H_3B_), 3.21–3.11 (m, 2H, H_10_), 2.12–1.05 (m, 1H, H_5A_), 1.85–1.73 (m, 2H, H_4_), 1.29–1.10 (m, 1H, H_5B_) ppm; **^13^C NMR (126 MHz, MeOD-*d*_4_)** δ 170.9 (C=O), 166.9 (C=O), 137.3 (C*_arom_*), 131.1 (CH*_arom_*), 129.5 (CH*_arom_*), 128.1 (CH*_arom_*), 60.1 (CH), 57.7 (CH), 46.0 (CH_2_), 38.2 (CH_2_), 29.4 (CH_2_), 22.8 (CH_2_) ppm; **HRMS (ESI+)** *m*/*z* calculated for C_14_H_17_N_2_O_2_ [M+H]^+^ 245.1285 was found to be 245.1283. Literature pertaining to NMR was reported for CDCl_3_ [21].

Cyclo-(d-Phe-l-Pro): HPLC elution time was 36 min. **^1^H NMR (500 MHz, MeOD-*d*_4_)** δ 7.33–7.28 (m, 3H, ArH), 7.19 (dd, *J* = 6.6, 2.9 Hz, 2H, ArH), 4.20 (td, *J* = 4.7, 1.0 Hz, 1H, H_9_), 3.56–3.51 (m, 1H, H_3A_), 3.36–3.31 (m, 1H, H_3B_), 3.20 (dd, *J* = 13.6, 4.8 Hz, 1H, H_10A_), 2.99 (dd, *J* = 13.7, 4.7 Hz, 1H, H_10B_), 2.61 (dd, *J* = 10.7, 6.3 Hz, 1H, H_6_), 2.08–2.00 (m, 1H, H_5A_), 1.96–1.86 (m, 1H, H_4A_), 1.73–1.55 (m, 2H, H_4B_, H_5B_) ppm; **^13^C NMR (126 MHz, MeOD-*d*_4_)** δ 171.3 (C=O), 167.4 (C=O), 136.7 (C*_arom_*), 131.3 (CH*_arom_*), 129.7 (CH*_arom_*), 128.5 (CH*_arom_*), 59.7 (CH), 59.1 (CH), 46.1 (CH_2_), 41.0 (CH_2_), 29.8 (CH_2_), 22.5 (CH_2_) ppm; **HRMS (ESI+)** *m*/*z* calculated for C_14_H_17_N_2_O_2_ [M+H]^+^ 245.1285 was found to be 245.1279. Literature pertaining to NMR was reported for CDCl_3_ [21].

### 4.4. Effect of Culturing Conditions and Comparison of Culture Extracts with Synthetic Standards by LCMS

Cultures of the *Bacillus* strain were grown in TCOATSS media (50 mL, in triplicate) in the presence of Diaion HP-20 resin (5% *w*/*v*). The resin used was untreated, sterilised by autoclave with media, or sterilised by UV exposure (for 2 h). All cultures were grown as detailed in Section 4.2. The cell pellets and resin were collected by centrifugation (10 min, 4000× *g*), followed by extraction with 20 mL of methanol for 30 min. The methanolic extract was then filtered, diluted 1:10 in 50% methanol–water, and analysed by LCMS.

### 4.5. Synthetic Procedures and Other Methods

#### 4.5.1. Preparation of l-Phenylalanine Methyl Ester Hydrochloride (**2**)

Acetyl chloride (5.4 mL, 75.0 mmol, 2.5 eq.) was added to a flask containing a solution of l-phenylalanine (1) (5.0 g, 30.3 mmol, 1 eq.) dissolved in dry methanol (~40 mL) at 0 °C under nitrogen. The reaction was stirred overnight while allowing it to warm to r.t. The solvent was removed *in vacuo* to produce l-phenylalanine methyl ester hydrochloride (2) (6.1 g, 93%), a white hydrochloride salt that was used without further purification. m.p. 160–162 °C [lit. 158–159 °C]; [α]^20^_D_ = +33.0 (c = 1.0, EtOH) [lit. +32.5 (c = 2.0, EtOH)] [37]; ^1^H NMR (500 MHz, D_2_O) δ 7.44–7.35 (m, 3H, Ar), 7.29–7.25 (m, 2H, Ar), 4.41 (dd, J = 7.5, 5.9 Hz, 1H, C_α_H), 3.82 (s, 3H, CH_3_), 3.32 (dd, J = 14.5, 5.9 Hz, 1H, C_β_H_A_), 3.21 (dd, J = 14.5, 7.5 Hz, 1H, C_β_H_B_) ppm; ^13^C NMR (126 MHz, D_2_O) δ 170.0 (C=O), 133.6 (C_arom_), 129.4 (CH_arom_), 129.2 (CH_arom_), 128.1 (CH_arom_), 54.1 (C_α_H), 53.5 (CH_3_), 35.5 (C_β_H_2_) ppm; HRMS (ESI+) *m*/*z* calculated for C_10_H_14_NO_2_ [M+H]^+^ 180.1019 was found to be 180.1016.

#### 4.5.2. Preparation of Boc-l-Proline (**3**)

A solution of di-*t*-butyl dicarbonate (480 mg, 2.2 mmol, 1.1 eq.) in dioxane (~5 mL) was added to a flask containing a solution of proline (230 mg, 2.0 mmol, 1 eq.) dissolved in water (~5 mL) at 0 °C. Aqueous KOH (4.5 M, 0.5 mL, 1.1 eq.) was added dropwise. The mixture was stirred overnight while warming it to r.t. The solution was concentrated on a rotary evaporator to remove the dioxane. Then, it was diluted with water (8 mL) and extracted with a mixture of diethyl ether and hexane (1:1) (3 × 10 mL). The aqueous layer was cooled by adding a small amount of ice and the pH was adjusted to 2 using 1 M HCl. The resultant white suspension was extracted using ethyl acetate (5 × 10 mL). The combined organic layers were dried over anhydrous MgSO_4_, and the solvent was removed *in vacuo* to produce a crude Boc-protected amino acid that was used without further purification.

The above procedure produced 275 mg (64% yield from l-proline) of the desired Boc-l-proline (**3**) as a white solid. **mp** 130–132 °C [lit. 133 °C]; **[*α*]^20^_D_** = −93.0 (c = 1.0, CHCl_3_) [lit. −94.5 (c = 0.75, CHCl_3_)] [27]; **^1^H NMR (500 MHz, CDCl_3_, mixture of rotamers)** δ 4.35 (d, *J* = 6.6 Hz, 0.6H, C*_α_*H), 4.25 (s, 0.4H, C*_α_*H), 3.61–3.27 (m, 2H, CH*_E_*H*_F_*), 2.32 (m, 1H, C*_β_*H*_A_*), 2.09–1.83 (m, 3H, C*_β_*H*_BCD_*), 1.49 (s, 5.4H, CH_3_), 1.42 (s, 3.6H, CH_3_) ppm; **^13^C NMR (126 MHz, CDCl_3_, mixture of rotamers)** δ 178.6 (C=O), 176.7 (C=O), 155.6 (C=O), 154.1 (C=O), 80.8 (C), 80.5 (C), 59.0 (C*_α_*H), 58.9 (C*_α_*H), 46.8 (CH_2_), 46.4 (CH_2_), 30.9 (CH_2_), 29.3 (CH_2_), 28.4 (CH_3_), 28.3 (CH_3_), 24.3 (CH_2_), 23.7 (CH_2_) ppm; **HRMS (ESI+)** *m*/*z* calculated for C_10_H_17_NO_4_Na [M+Na]^+^ 238.1050 was found to be 238.1041.

#### 4.5.3. Peptide Coupling Using Protected Amino Acids

General procedure: Boc-proline (**3**) (500 mg, 2.3 mmol, 1 eq.), Phe-OMe.HCl (**2**) (496 mg, 2.3 mmol, 1 eq.), EDC.HCl (529 mg, 2.7 mmol, 1.2 eq.), and HOBt (311 mg, 2.7 mmol, 1.2 eq.) were dissolved in DCM (~20 mL) at 0 °C. The reaction mixture was stirred for 5 min and then Et_3_N (0.6 mL, 2 eq.) was added dropwise. The reaction mixture was stirred for 20 h while it warmed to r.t. The reaction mixture was concentrated *in vacuo* then diluted with ethyl acetate (50 mL) and washed successively with water (1 × 50 mL), aqueous citric acid (5%, 4 × 15 mL), and aqueous potassium carbonate (10%, 2 × 25 mL). The combined organic layers were dried over anhydrous MgSO_4_, and the solvent was removed *in vacuo*. Purification by column chromatography using silica gel (gradient hexane–ethyl acetate, 10–100%) produced Boc-Pro-Phe-OMe (**4**) as a white solid.

#### 4.5.4. Boc-l-Pro-l-Phe-OMe (**4**)

The above procedure produced 590 mg of Boc-l-Pro-l-Phe-OMe (**4**) (85% yield from Boc-l-Pro (**3**) and l-Phe-OMe (**2**). **mp** 71–73 °C [lit. 76–78 °C] [38]; **[*α*]^20^_D_** = −54.3 (c = 1.0, CHCl_3_) [lit. −54.1 (c = 1.0, CHCl_3_)] [39]; **^1^H NMR (500 MHz, DMSO-*d*_6_, mixture of rotamers)** δ 8.22 (d, *J* = 7.9 Hz, 0.6H, NH), 8.18 (d, *J* = 7.5 Hz, 0.3H, NH), 7.30–7.15 (m, 5H, ArH), 4.57–4.49 (m, 0.7H, CH*_Phe_*), 4.45 (q, *J* = 7.4 Hz, 0.3H, CH*_Phe_*), 4.12 (d, *J* = 7.8 Hz, 0.3H, CH*_Pro_*), 4.01 (dd, *J* = 8.6, 3.4 Hz, 0.7H, CH*_Pro_*), 3.61 (s, 2H, OCH_3_), 3.56 (s, 1H, OCH_3_), 3.37–3.30 (m, expected 1H, overlaps with water, CH*_EPro_*), 3.27–3.18 (m, 1H, CH*_FPro_*), 3.09–2.89 (m, 2H, CH*_APhe_*H*_BPhe_*), 2.07–1.95 (m, 1H, CH*_APro_*), 1.76–1.54 (m, 3H, CH*_BPro_*H*_CPro_*H*_DPro_*), 1.38 (s, 2.6H, CCH_3_), 1.21 (s, 6.4H, CCH_3_) ppm; **^13^C NMR (126 MHz, DMSO-*d*_6_, mixture of rotamers)** δ 172.6 (C=O), 172.3 (C=O), 172.1 (C=O), 171.9 (C=O), 153.7 (C=O), 153.3 (C=O), 137.4 (C*_arom_*), 137.2 (C*_arom_*), 129.2 (CH*_arom_*), 129.0 (CH*_arom_*), 128.2 (CH*_arom_*), 126.5 (CH*_arom_*), 78.6 (C), 78.4 (C), 59.5 (CH), 59.2 (CH), 53.5 (CH), 53.3 (CH), 51.9 (OCH_3_), 51.8 (OCH_3_), 46.6 (CH_2_), 46.4 (CH_2_), 36.6 (CH_2_), 36.4 (CH_2_), 30.8 (CH_2_), 29.6 (CH_2_), 28.2 (CCH_3_), 27.8 (CCH_3_), 23.7 (CH_2_), 22.9 (CH_2_) ppm; **HRMS (ESI+)** *m*/*z* calculated for C_20_H_29_N_2_O_5_ [M+H]^+^ 377.2071 was found to be 377.2063.

#### 4.5.5. Boc-d-Pro-d-Phe-OMe

The above procedure produced Boc-d-Pro-d-Phe-OMe (75% yield from Boc-d-Pro and d-Phe-OMe). **mp** 73–75 °C; **[*α*]^20^_D_** = +50.0 (c = 1.0, CHCl_3_); **^1^H NMR (500 MHz, DMSO-*d*_6_, mixture of rotamers)** δ 8.23 (d, *J* = 7.9 Hz, 0.7H, NH), 8.18 (d, *J* = 7.4 Hz, 0.3H, NH), 7.31–7.15 (m, 5H, ArH), 4.55–4.41 (m, 1H, CH*_Phe_*), 4.12 (d, *J* = 7.6 Hz, 0.3H, CH*_Pro_*), 4.01 (dd, *J* = 8.6, 3.4 Hz, 0.7H, CH*_Pro_*), 3.61 (s, 2H, OCH_3_), 3.56 (s, 1H, OCH_3_), 3.34–3.30 (m, 1H, CH*_Pro_*), 3.25–3.18 (m, 1H, CH*_Pro_*), 3.10–2.98 (m, 1H, CH*_APhe_*), 2.98–2.89 (m, 1H, CH*_BPhe_*), 2.05–1.88 (m, 1H, CH*_Pro_*), 1.76–1.62 (m, 2H, CH*_Pro_*H*_Pro_*), 1.59–1.53 (m, 1H, CH*_Pro_*), 1.38 (s, 3H, CCH_3_), 1.21 (s, 6H, CCH_3_) ppm; **^13^C NMR (126 MHz, DMSO-*d*_6_, mixture of rotamers)** δ 172.7 (C=O), 172.1 (C=O), 153.7 (C=O), 153.3 (C=O), 137.4 (C*_arom_*), 137.2 (C*_arom_*), 129.2 (CH*_arom_*), 129.0 (CH*_arom_*), 128.2 (CH*_arom_*), 126.5 (CH*_arom_*), 78.7 (C), 78.4 (C), 59.6 (CH), 59.2 (CH), 53.6 (CH), 53.4 (CH), 51.9 (OCH_3_), 51.8 (OCH_3_), 46.7 (CH_2_), 46.5 (CH_2_), 36.6 (CH_2_), 36.4 (CH_2_), 30.8 (CH_2_), 29.7 (CH_2_), 28.2 (CCH_3_), 27.9 (CCH_3_), 23.7 (CH_2_), 22.9 (CH_2_) ppm; **HRMS (ESI+)** *m*/*z* calculated for C_20_H_29_N_2_O_5_ [M+H]^+^ 377.2071 was found to be 377.2069.

#### 4.5.6. Boc-l-Pro-d-Phe-OMe

The above procedure produced Boc-l-Pro-d-Phe-OMe (77% yield from Boc-l-Pro and d-Phe-OMe). **mp** 100–102 °C; **[*α*]^20^_D_** = −86.0 (c = 1.0, CHCl_3_); **^1^H NMR (500 MHz, DMSO-*d*_6_, mixture of rotamers)** δ 8.34 (d, *J* = 8.3 Hz, 0.7H, NH), 8.20 (d, *J* = 8.2 Hz, 0.3H, NH), 7.33–7.13 (m, 5H, ArH), 4.64–4.52 (m, 1H, CH*_Phe_*), 4.08 (d, *J* = 8.3 Hz, 0.3H, CH*_Pro_*), 4.04 (dd, *J* = 8.3, 3.6 Hz, 0.7H, CH*_Pro_*), 3.64 (s, 1H, OCH_3_), 3.61 (s, 2H, OCH_3_), 3.26 (m, 1H, CH*_Pro_*), 3.24–3.15 (m, 1H, CH*_Pro_*), 3.08 (m, 1H, CH*_APhe_*), 2.95–2.84 (m, 1H, CH*_BPhe_*), 2.00–1.84 (m, 1H, CH*_Pro_*), 1.70–1.53 (m, 2H, CH*_Pro_*H*_Pro_*), 1.50–1.43 (m, 1H, CH*_Pro_*), 1.39 (s, 3H, CCH_3_), 1.28 (s, 6H, CCH_3_) ppm; **^13^C NMR (126 MHz, DMSO-*d*_6_, mixture of rotamers)** δ 172.3 (C=O), 172.0 (C=O), 153.6 (C=O), 153.3 (C=O), 137.2 (C*_arom_*), 129.2 (CH*_arom_*), 129.1 (CH*_arom_*), 128.2 (CH*_arom_*), 126.5 (CH*_arom_*), 78.5 (C), 78.4 (C), 59.3 (CH), 59.2 (CH), 53.0 (CH), 52.8 (CH), 52.0 (OCH_3_), 51.8 (OCH_3_), 46.6 (CH_2_), 46.4 (CH_2_), 36.9 (CH_2_), 30.9 (CH_2_), 30.0 (CH_2_), 28.2 (CCH_3_), 27.9 (CCH_3_), 23.6 (CH_2_), 22.9 (CH_2_) ppm; **HRMS (ESI+)** *m*/*z* calculated for C_20_H_29_N_2_O_5_ [M+H]^+^ 377.2071 was found to be 377.2068.

#### 4.5.7. Boc-d-Pro-l-Phe-OMe

The above procedure produced Boc-d-Pro-l-Phe-OMe (73% yield from Boc-d-Pro and l-Phe-OMe). **mp** 102–104 °C; **[*α*]^20^_D_** = +83.4 (c = 1.0, CHCl_3_); **^1^H NMR (500 MHz, DMSO-*d*_6_, mixture of rotamers)** δ 8.34 (d, *J* = 8.3 Hz, 0.7H, NH), 8.20 (d, *J* = 8.3 Hz, 0.3H, NH), 7.31–7.11 (m, 5H, ArH), 4.62–4.52 (m, 1H, CH*_Phe_*), 4.11–4.05 (m, 0.3H, CH*_Pro_*), 4.04 (dd, *J* = 6.8, 3.1 Hz, 0.7H, CH*_Pro_*), 3.64 (s, 1H, OCH_3_), 3.61 (s, 2H, OCH_3_), 3.31–3.26 (m, 1H, CH*_Pro_*), 3.24–3.18 (m, 1H, CH*_Pro_*), 3.10–3.05 (m, 1H, CH*_APhe_*), 2.95–2.84 (m, 1H, CH*_BPhe_*), 1.97–1.88 (m, 1H, CH*_Pro_*), 1.67–1.52 (m, 2H, CH*_Pro_*H*_Pro_*), 1.50–1.43 (m, 1H, CH*_Pro_*), 1.39 (s, 3H, CCH_3_), 1.28 (s, 6H, CCH_3_) ppm; **^13^C NMR (126 MHz, DMSO-*d*_6_, mixture of rotamers)** δ 172.3 (C=O), 172.0 (C=O), 153.6 (C=O), 153.3 (C=O), 137.2 (C*_arom_*), 129.2 (CH*_arom_*), 129.1 (CH*_arom_*), 128.2 (CH*_arom_*), 126.5 (CH*_arom_*), 78.5 (C), 78.4 (C), 59.3 (CH), 59.3 (CH), 53.0 (CH), 52.8 (CH), 52.0 (OCH_3_), 51.8 (OCH_3_), 46.6 (CH_2_), 46.4 (CH_2_), 36.9 (CH_2_), 31.0 (CH_2_), 30.0 (CH_2_), 28.2 (CCH_3_), 27.9 (CCH_3_), 23.6 (CH_2_), 22.9 (CH_2_) ppm; **HRMS (ESI+)** *m*/*z* calculated for C_20_H_29_N_2_O_5_ [M+H]^+^ 377.2071 was found to be 377.2068.

#### 4.5.8. Boc Deprotection

General procedure: The reaction was carried out in a microwave vial. Water (0.2 mL) and TFA (0.4 mL, excess) were added to a solution of Boc-Pro-Phe-OMe (200 mg, 0.5 mmol) in DCM (2 mL) at 0 °C. The reaction vial was sealed and heated in a microwave reactor at 60 °C for 40 min. The reaction mixture was cooled and the solvents were evaporated *in vacuo.* The crude product was washed with diethyl ether. The compound was then used without further purification.

#### 4.5.9. l-Pro-l-Phe-OMe·TFA (**5**)

The above procedure produced l-Pro-l-Phe-OMe·TFA (**5**) from (**4**). The crude product was used without any purification. **mp** 74–76 °C [lit. 70–74 °C] [40]; **^1^H NMR (500 MHz, MeOD-*d*_4_)** δ 7.31–7.21 (m, 5H), 4.70 (dd, *J* = 9.4, 5.4 Hz, 1H), 4.24–4.20 (m, 1H), 3.67 (s, 3H), 3.38–3.36 (m, 1H), 3.29–3.26 (m, 1H), 3.22 (dd, *J* = 14.0, 5.4 Hz, 1H), 3.00 (dd, *J* = 14.1, 9.5 Hz, 1H), 2.41–2.35 (m, 1H), 2.05–1.99 (m, 3H) ppm; **^13^C NMR (126 MHz, MeOD-*d*_4_)** δ 172.9, 169.8, 138.0, 130.1, 129.6, 128.0, 60.8, 55.6, 52.9, 47.4, 38.0, 31.0, 24.9 ppm; **HRMS (ESI+)** *m*/*z* calculated for C_15_H_21_N_2_O_3_ [M+H]^+^ 277.1547 was found to be 277.1532.

#### 4.5.10. d-Pro-d-Phe-OMe·TFA

The above procedure produced d-Pro-d-Phe-OMe·TFA. The crude product was used without any purification. **^1^H NMR (500 MHz, MeOD-*d*_4_)** δ 7.30–7.19 (m, 5H), 4.72 (dd, *J* = 9.4, 5.3 Hz, 1H), 4.25–4.19 (m, 1H), 3.71 (s, 3H), 3.38–3.35 (m 1H), 3.30–3.25 (m, 1H), 3.22 (dd, *J* = 14.0, 5.4 Hz, 1H), 3.00 (dd, *J* = 14.0, 9.4 Hz, 1H), 2.45–2.36 (m, 1H), 2.09–1.94 (m, 3H) ppm; **^13^C NMR (126 MHz, MeOD-*d*_4_)** δ 172.9, 169.8, 138.0, 132.4, 130.1, 129.6, 128.0, 60.8, 55.6, 52.9, 47.4, 38.0, 31.0, 24.9 ppm; **HRMS (ESI+)** *m*/*z* calculated for C_15_H_21_N_2_O_3_ [M+H]^+^ 277.1547 was found to be 277.1539.

#### 4.5.11. l-Pro-d-Phe-OMe·TFA

The above procedure produced l-Pro-d-Phe-OMe·TFA. The crude product was used without any purification. **^1^H NMR (500 MHz, MeOD-*d*_4_)** δ 7.31–7.26 (m, 2H), 7.25–7.18 (m, 3H), 4.81 (ddd, *J* = 17.3, 10.3, 4.8 Hz, 1H), 4.23 (dd, *J* = 8.3, 6.4 Hz, 1H), 3.74 (s, 2H*), 3.25–3.21 (m, 3H), 2.92 (ddd, *J* = 14.1, 10.3, 2.4 Hz, 1H), 2.24–2.14 (m, 1H), 1.92–1.82 (m, 1H), 1.75–1.66 (m, 1H), 1.57–1.51 (m, 1H) ppm; **^13^C NMR (126 MHz, MeOD-*d*_4_)** δ 174.2, 169.5, 137.9, 130.3, 129.5, 128.0, 60.9, 54.9, 52.9, 47.2, 38.5, 31.1, 24.7 ppm; **HRMS (ESI+)** *m*/*z* calculated for C_15_H_21_N_2_O_3_ [M+H]^+^ 277.1547 was found to be 277.1538. *A lower integration was observed for methyl protons in ^1^H NMR of the crude sample.

#### 4.5.12. d-Pro-l-Phe-OMe·TFA

The above procedure produced d-Pro-l-Phe-OMe·TFA. The crude product was used without any purification. **^1^H NMR (500 MHz, MeOD-*d*_4_)** δ 7.32–7.26 (m, 2H), 7.25–7.19 (m, 3H), 4.83 (dd, *J* = 10.2, 5.0 Hz, 1H), 4.23 (dd, *J* = 8.6, 6.4 Hz, 1H), 3.74 (s, 2H*), 3.30–3.19 (m, 3H), 2.92 (ddd, *J* = 14.5, 10.3, 4.5 Hz, 1H), 2.25–2.14 (m, 1H), 1.95–1.84 (m, 1H), 1.77–1.67 (m, 1H), 1.60–1.50 (m, 1H) ppm; **^13^C NMR (126 MHz, MeOD-*d*_4_)** δ 174.5, 169.4, 137.9, 130.3, 129.5, 128.0, 60.9, 54.9, 52.9, 47.2, 38.5, 31.1, 24.7 ppm; **HRMS (ESI+)** *m*/*z* calculated for C_15_H_21_N_2_O_3_ [M+H]^+^ 277.1547 was found to be 277.1540. *A lower integration was observed for methyl protons in ^1^H NMR of the crude sample.

Descriptions of additional experimental and general methods, as well as LCMS and NMR characterisation data and images can be found in the Supplementary Materials.

## Figures and Tables

**Table 1:**
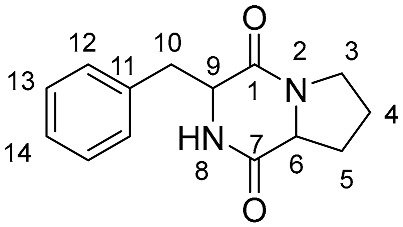
^1^H NMR comparison of the synthetic and the isolated natural cyclo-(Phe-Pro).

Position (Group) *	Synthetic *Trans* (l*,*d)	Synthetic *Cis* (l*,*l)	Natural Isolate
3 (CH_2_)	3.53 and 3.32	3.54 and 3.37	3.55 and 3.38
4 (CH_2_)	1.88 and 1.65	1.80	1.81
5 (CH_2_)	2.03 and 1.65	2.10 and 1.20	2.11 and 1.22
6 (CH)	2.60	4.07	4.08
9 (CH)	4.21	4.45	4.46
10 (CH_2_)	3.19 and 3.00	3.17	3.18
12–14 (Ar-H)	7.34–7.27 and 7.21–7.16	7.31–7.19	7.30–7.23

* Assignments are based on 2D NMRs (COSY, HSQC, HMBC) of synthetic isomers. Chemical shifts (δ ppm) are reported for NMR in MeOD-*d*_4_.

## Data Availability

Data are contained within the article and the Supplementary Materials. Analytical data included in this study are available from the corresponding author upon reasonable request.

## Supplementary Materials

The following supporting information can be downloaded at https://www.mdpi.com/article/10.3390/md23060234/s1. Table S1: Yield from the cyclisation depending on the solvent and base; Table S2: Summary of the cyclisation reactions and relative quantification using LCMS; Table S3: Summary of the stability tests using crude DKP mixture; Table S4: Summary of the deuteration using purified DKP.
