# Supplementary material for: Isolation and Identification of Cis-2,5-Diketopiperazine from a Novel Bacillus Strain and Synthesis of Its Four Stereoisomers
Source: Mar Drugs. 2025 May 29;23(6):234. doi: 10.3390/md23060234 (PMC12193752; doi:10.3390/md23060234)
Supplement: Supplementary file 1 [file marinedrugs-23-00234-s001.zip › marinedrugs-3647006-supplementary.pdf]

Isolation and identification of *cis*-2,5-diketopiperazine from a novel *Bacillus* strain and synthesis of its 4 stereoisomers

Alan M.C. Obled, Refaat B. Hamed, Edward Spence, Marija K. Zacharova, Sunil V. Sharma, Yunpeng Wang, Rosemary Lynch, Helen Connaris, Adina Tatheer, Marie-Lise Bourguet-Kondracki, Gordon J. Florence and Rebecca J.M. Goss *

Index

General methods 2

Characterisation of natural isolated cyclo-(Phe-Pro) 3

Screening of cyclisation conditions for synthesis 5

Optimisation of DKP cyclisation conditions, effect on epimerisation and stability of cyclo-(Phe-Pro) 6

Deuteration of DKP 7

NMR spectra of synthetic compounds and DKPs 9

General methods

All the chemicals, reagents and solvents were purchased from commercial suppliers (Acros, Alfa Aesar, Fluorochem or Sigma Aldrich) and used as received without further purification. ^{1}H NMR and ^{13}C NMR were run on Bruker instruments (Ascend^{TM} at 500 MHz for ^{1}H and 126 MHz for ^{13}C). For ^{1}H NMR, chemical shifts are referenced to deuterated solvent signals $CDCl_3$ (7.26 ppm), DMSO-d_6 (2.50 ppm), D_2O (4.79 ppm) or MeOD-d_4 (3.31 ppm) and reported as ppm shift from a hypothetical TMS signal. Coupling constants are reported in Hertz (Hz). Multiplicities are reported as: s = singlet, d = doublet, t = triplet, q = quartet, m = multiplet, and combinations of these symbols. Carbon spectra (decoupled from ^{1}H) were recorded with multiplicity editing using a DEPTQ pulse sequence. For ^{13}C NMR, chemical shifts are referenced to deuterated solvent signals $CDCl_3$ (77.2 ppm), DMSO-d_6 (39.5 ppm) or MeOD-d_4 (49.0 ppm) and reported as ppm shift from a hypothetical TMS signal. 2D NMR techniques (COSY, HSQC, HMBC) were used to confirm assignments.

Low- and High-resolution mass spectra were recorded on a Waters Micromass LCT time of flight mass spectrometer coupled to a Waters 2975 HPLC system or an Orbitrap ELOS pro. Data analysis was performed with Thermo Xcalibur 3.0.63.3 software. Samples were prepared in a 1:1 mixture of methanol water and injected with a 100 µL syringe. The solvent system consists of 0.1% formic acid in water (A) and acetonitrile (B). Products were eluted using a standard gradient from 5% B to 100% B over 15 min with a flow rate of 0.35 mL/min. The column used was a Kinetex® 2.6 µm EVO C18 100 Å, LC Column 100 x 2.1 mm. The exact masses were calculated using Chemdraw. The peak area for the exact mass of a target molecule correspond to the area under a peak related to the calculated exact mass ±0.001.

HPLC purification was carried out on a Gilson HPLC system (Gilson 322 pump, Gilson UV/Vis-151 detector, Gilson 402 syringe pump). The column used was an XBridge Semi-Prep Phenyl 5 µm column (10 x 250 mm). Samples were prepared in a 1:1 mixture of methanol water. The solvent system consists of 0.1% formic acid in water (A) and methanol (B). Products were eluted using a standard gradient from 5% B to 60% B over 50 min, 60% B to 98% B over 1 min, 98% B for 2 min with a flow rate of 4 mL/min.

TLC analysis was performed on commercial polyester sheets bearing 0.20mm layer of silica gel with fluorescent indicator UV_{254}. Flash chromatography was carried out using Biotage Isolera Four system, using prepacked cartridges containing either silica or reversed-phase C18 silica. The melting points (m.p.) were recorded using a Stuart® melting point SMP30. Optical activity (αD) was measured on a Perkin Elmer Model 341 polarimeter at a temperature of 20

°C using a Na/Hal lamp at 589 nm. Microwave reactions were carried out using Biotage Initiator+ in appropriate Biotage Microwave Vials sealed with aluminium crimp caps.

Characterisation of natural isolated cyclo-(Phe-Pro)

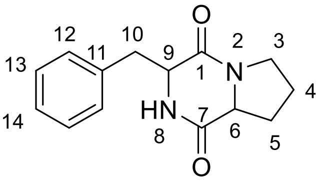

^{1}H NMR (700 MHz, MeOD-d_4) δ 7.30– 7.23 (m, 5H, Ar-H), 4.46 (td, J = 5.0, 1.9 Hz, 1H, H_9), 4.08 (ddd, J = 11.0, 6.4, 2.0 Hz, 1H, H_6), 3.55 (dt, J = 11.9, 8.3 Hz, 1H, H_{3A}), 3.42 – 3.36 (m, 1H, H_{3B}), 3.24 – 3.12 (m, 2H, H_{10}), 2.11 (dtd, J = 12.3, 6.1, 3.3 Hz, 1H, H_{5A}), 1.84 – 1.79 (m, 2H, H_4), 1.22 (td, J = 9.7, 8.8, 2.1 Hz, 1H, H_{5B}). **HRMS (ESI+)** *m/z* calculated for $C_{14}H_{17}N_2O_2$ $[M+H]^+$ 245.1285, found 245.1277.

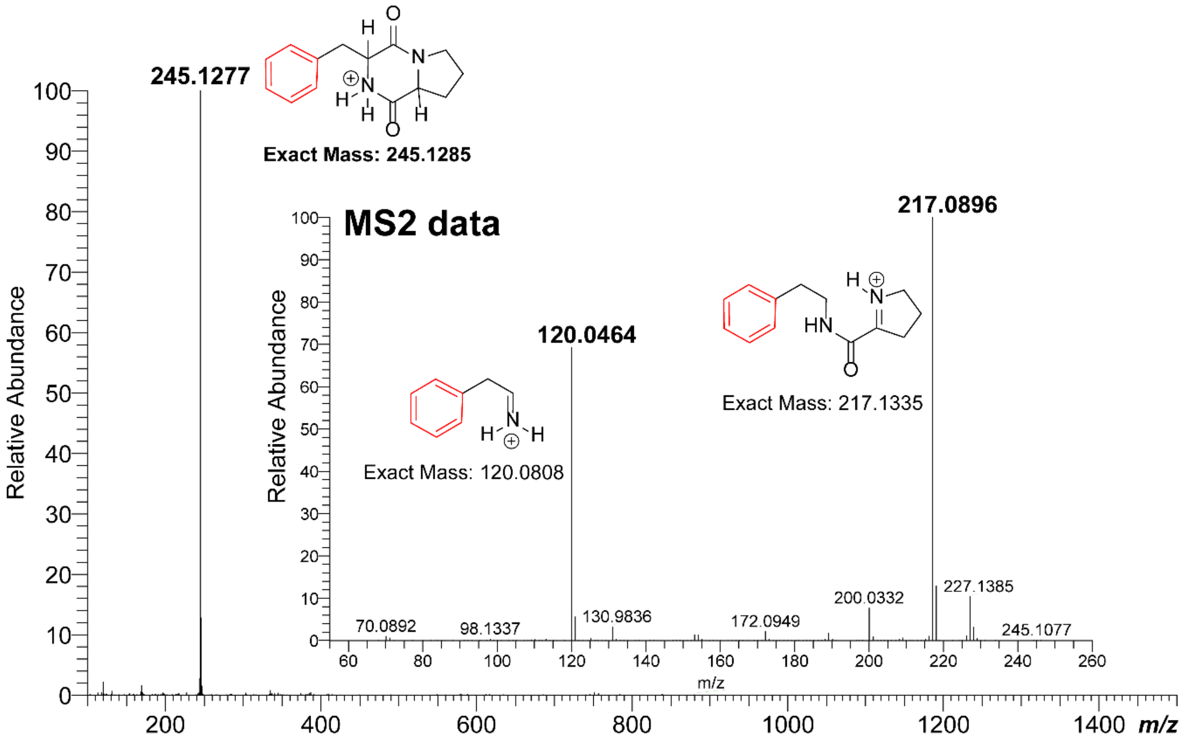

HRMS and MS2 data for natural isolated cyclo-(Phe-Pro)

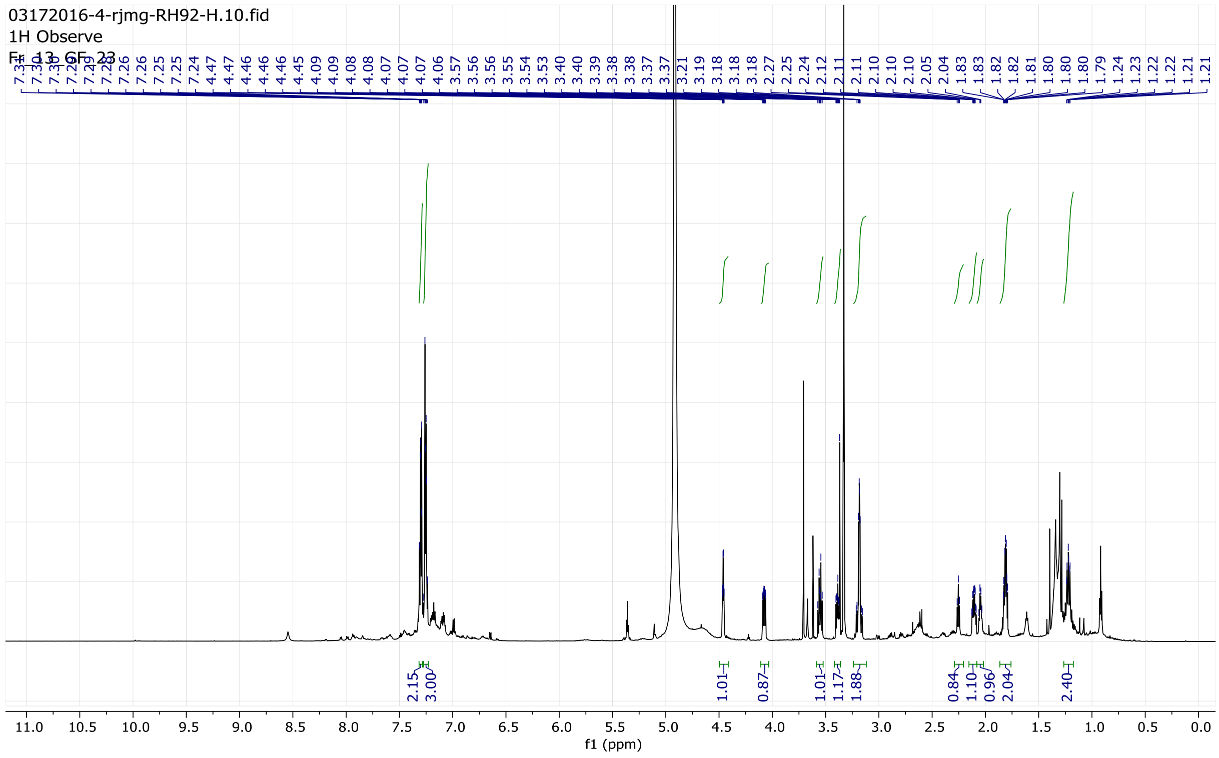

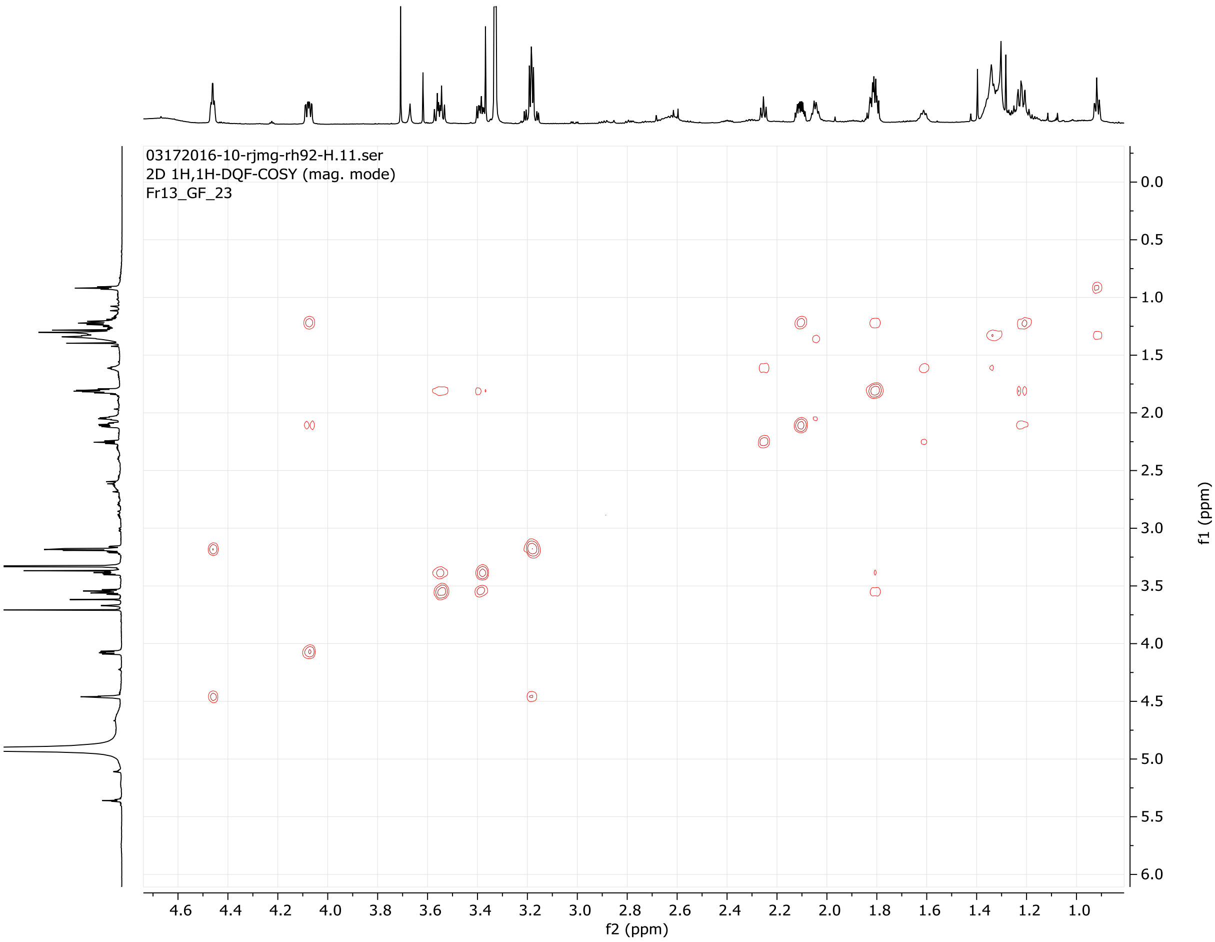

^{1}H NMR and COSY spectra of natural isolated cyclo-(Phe-Pro)

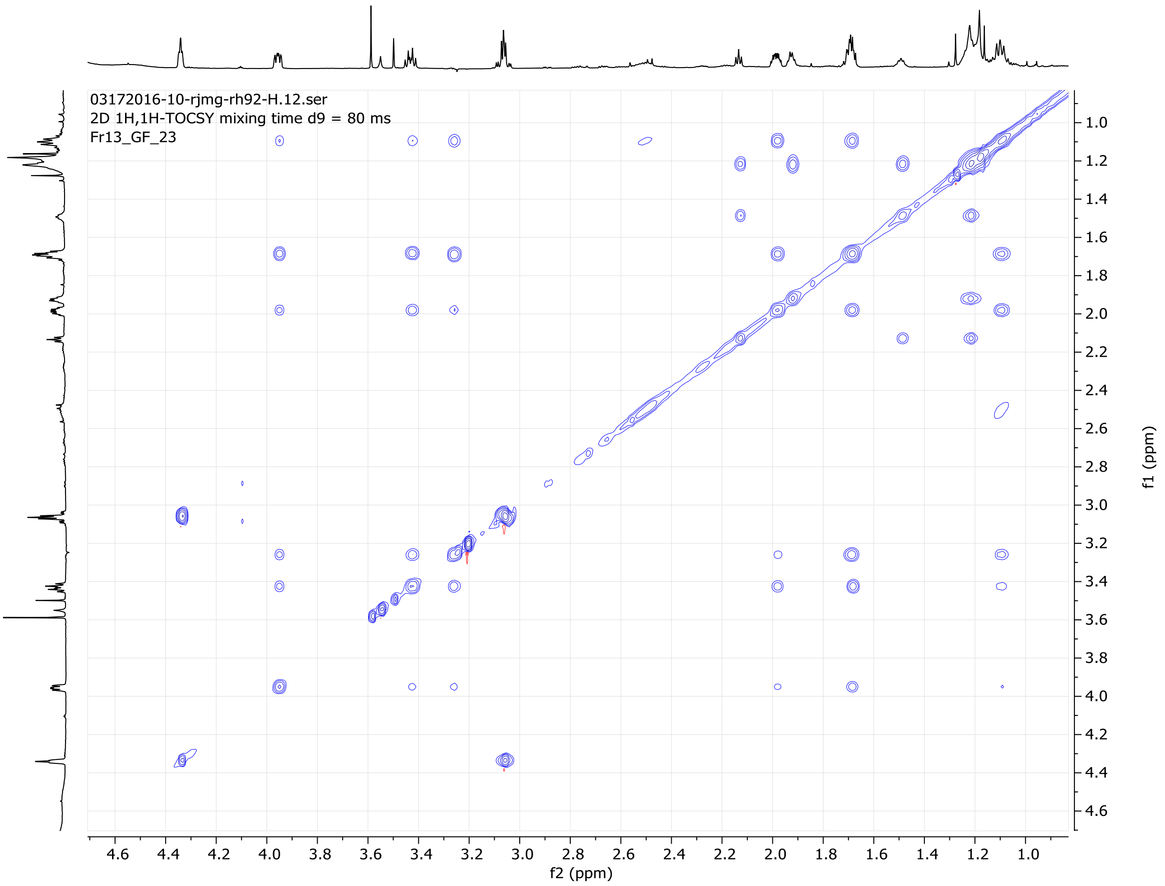

TOCSY spectrum of natural isolated cyclo-(Phe-Pro)

Screening of cyclisation conditions for synthesis

A solution of L-Pro-L-Phe-OMe·TFA (**5**, 0.2 mmol) and base (5 eq.) in appropriate solvent (water or methanol, 5 mL) were treated as given in **Table S-1**.

Table S-1: Yield from the cyclisation depending on the solvent and base

Entry	Solvent	Base	Temperature*	Yield (%)
**1**	Water	Na_2CO_3	r.t.	39
**2**	Water	Na_2CO_3	Reflux	72
**3**	Methanol	Na_2CO_3	Reflux	89
**4**	Methanol	Et_3N	Reflux	not isolated (>80% conversion)
**5**	Methanol	Et_3N	r.t.	55
**6**	Methanol	Na_2CO_3	MW (80 °C, 30 min)	93

*Overnight reactions except entry 6.

Optimisation of DKP cyclisation conditions, effect on epimerisation and stability of cyclo-(Phe-Pro)

Using the protocol for cyclisation (Na_2CO_3 in MeOH), the reactions were conducted using conventional reflux or microwave heating as given in Table S-2. At various time points, aliquots of reaction mixture were neutralised and frozen. Once all the samples were collected, they were warmed to r.t. and prepared for LC-MS analysis.

Table S-2: Summary of the cyclisation reactions and relative quantification using LCMS

Entry	Heating	Time	Temperature	Conversion (DKP %)	*Trans* DKP	*Cis* DKP*
**1**	Hot plate	20 mins	Reflux	1	0	1
**2**	Hot plate	35 mins	Reflux	19	0.23	0.77
**3**	Hot plate	1 h 45	Reflux	92	0.84	0.16
**4**	Hot plate	3 h	Reflux	99	0.86	0.14
**5**	Hot plate	4 h	Reflux	99	0.88	0.12
**6**	Hot plate	5 h	Reflux	100	0.88	0.12
**7**	Hot plate	21 h	Reflux	100	0.88	0.12
**8**	MW	10 mins	80 °C	41	0.56	0.44
**9**	MW	20 mins	80 °C	92	0.80	0.20
**10**	MW	30 mins	80 °C	93	0.75	0.25
**11**	MW	60 mins	80 °C	100	0.82	0.18
**12**	MW	30 mins	60 °C	38	0.37	0.63
**13**	MW	30 mins	100 °C	100	0.75	0.25
**14**	MW	30 mins	120 °C	100	0.77	0.23
**15**	MW	10 mins	120 °C	100	0.76	0.24

*LCMS analysis was used to determine ratio of *trans* or *cis* DKP (normalised data is presented).

Stability of DKP mixture was tested under different conditions (Table S-3). Sample solutions from the reaction (Na_2CO_3 in methanol) were used as such or pH modified using acetic acid. At various time points, LCMS analysis was used to compare the evolution of the *cis/trans* DKP ratio.

Table S-3: Summary of the stability tests using crude DKP mixture.

Entry	pH	Conditions	*Trans* DKP	*Cis* DKP*
**1**	Control sample	Starting composition	0.67	0.33
**2**	9 – 9.5	-20 °C, 4 days	0.66	0.34
**3**	9 – 9.5	r.t., 4 days	0.84	0.16
**4**	Neutral	-20 °C, 4 days	0.65	0.35
**5**	Neutral	r.t., 4 days	0.64	0.36
**6**	3 – 4	-20 °C, 4 days	0.64	0.36
**7**	3 – 4	r.t., 4 days	0.66	0.34
**8**	3 – 4	MW, 80 °C, 20 mins	0.64	0.36

*LCMS analysis was used to determine ratio of *trans* or *cis* DKP (normalised data is presented).

Deuteration of DKP

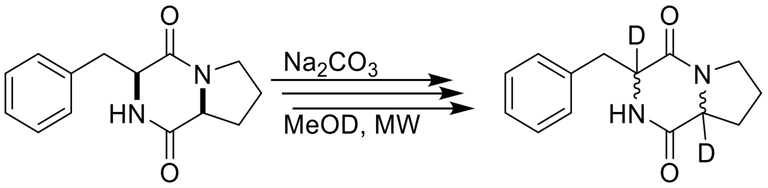

Purified samples of *trans* and *cis* cyclo-(Phe-Pro) were used for deuteration reaction. Purified diastereoisomer (1 equiv.) in MeOD-*d4* (1 mL) was treated with excess Na_2CO_3 (2 equiv.) and heated at 80 °C in a microwave. Samples were dried and analysed by ^{1}H NMR. We were able to confirm epimerisation on both the *cis* and *trans* purified cyclo-(Phe-Pro). ^{1}H NMR of the crude revealed almost complete deuteration of the CH_{Pro} and a lower degree of deuteration on the CH_{Phe}. LCMS analysis was used to calculate the extent of epimerisation along with ratio of mono-/di-deuterated cyclo-(Phe-Pro) (Table S-4). While deuterium exchange for NH proton is possible during the reaction, LCMS under aqueous condition reverts any exchange and tri-deuterated species were not observed on LCMS. *Cis* isomer showed 98% deuterium incorporation; whereas *trans* isomer showed only 85% deuteration under identical conditions. It is noteworthy that level epimerisation was significantly higher for *cis* isomer.

Table S-4: Summary of the deuteration using purified DKP.

Entry	Starting DKP	**Deuteration level** (composition determined by LCMS)								
		No Dueteration			Mono-deuterated DKP			Di-deuterated DKP		
		Trans	*Cis*	DKP (%)*	*Trans*	*Cis*	DKP (%)*	*Trans*	*Cis*	DKP (%)*
**1**	*Cis*	0.74	0.26	2%	0.85	0.15	58%	0.82	0.18	40%
**2**	*Trans*	0.96	0.04	15%	0.73	0.27	65%	0.64	0.36	20%

*Note the column DKP (%) represent the percent of the DKP at this level of deuteration in the reaction (*Trans* and *Cis* level combined).

NMR spectra of synthetic compounds

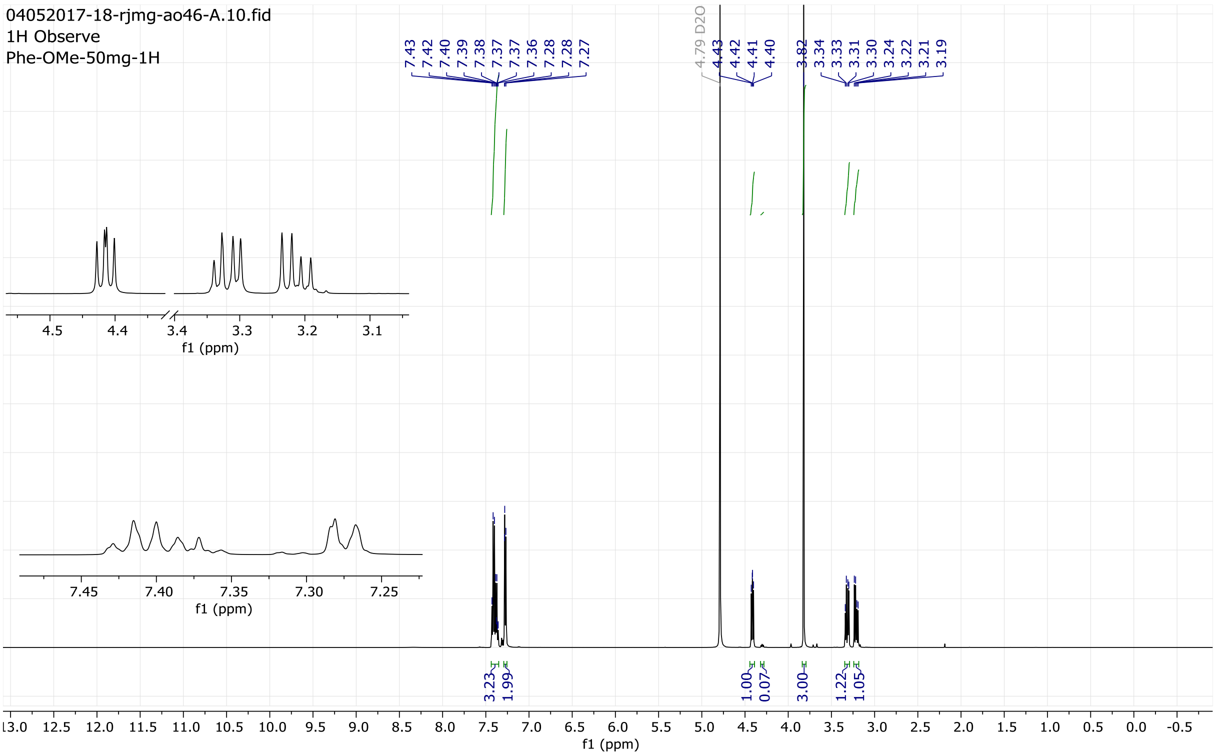

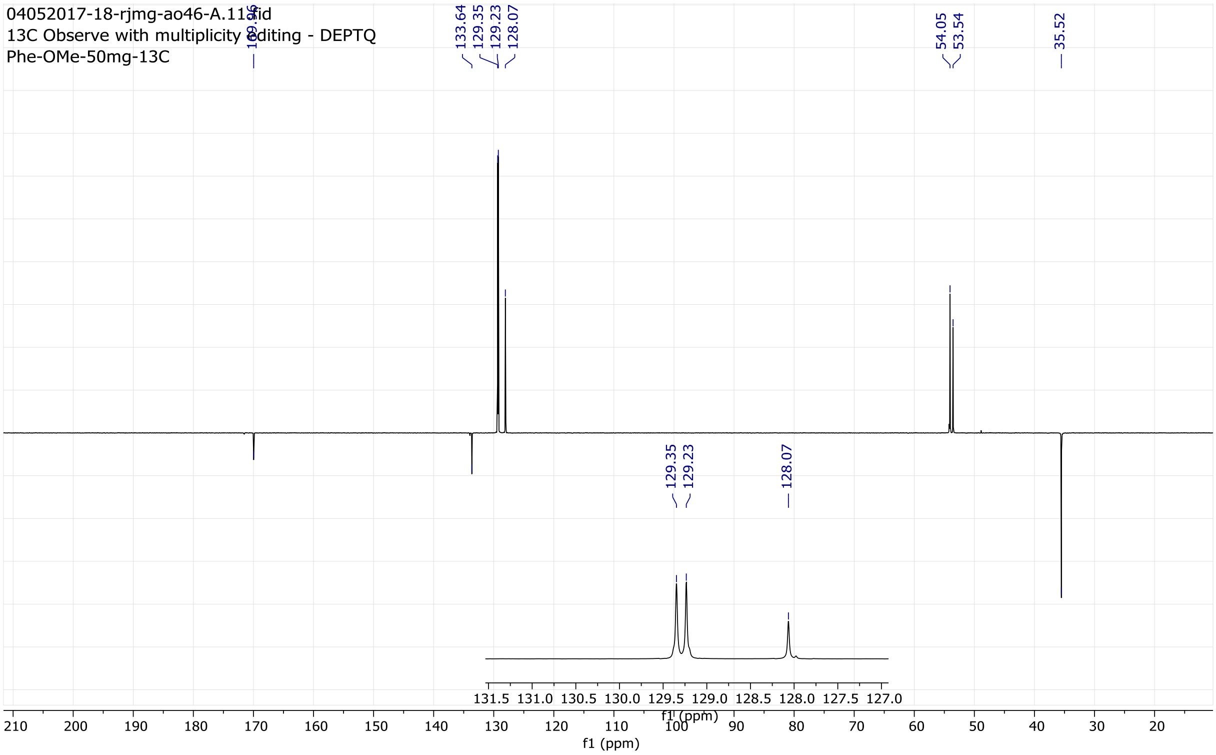

^1H and ^{13}C NMR of L-Phenylalanine methyl ester hydrochloride (2)

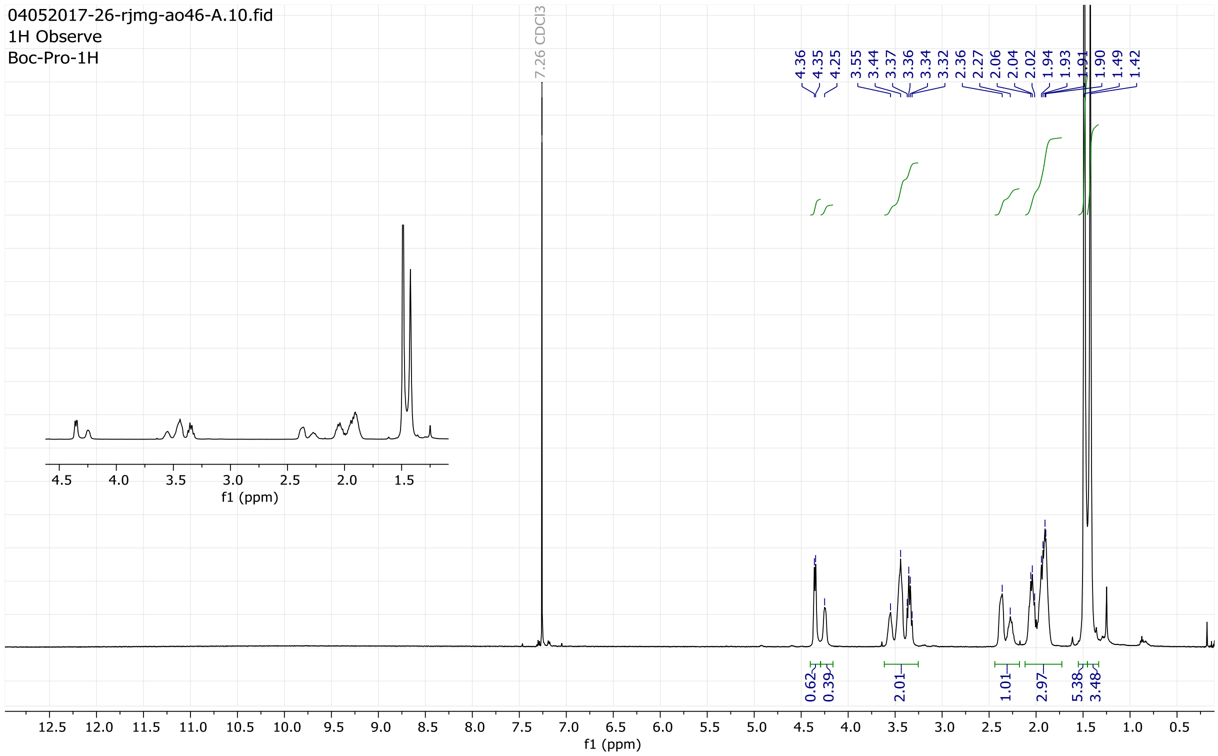

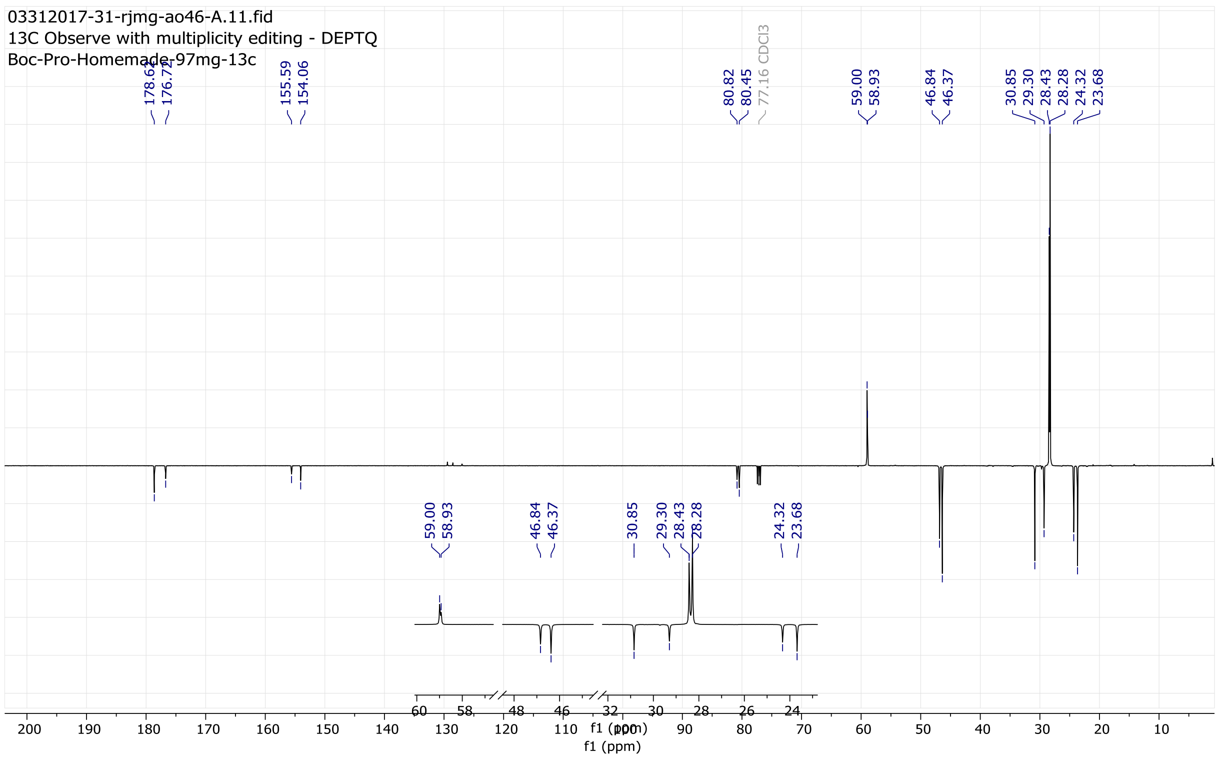

^{1}H and ^{13}C NMR of Boc-L-Proline (3)

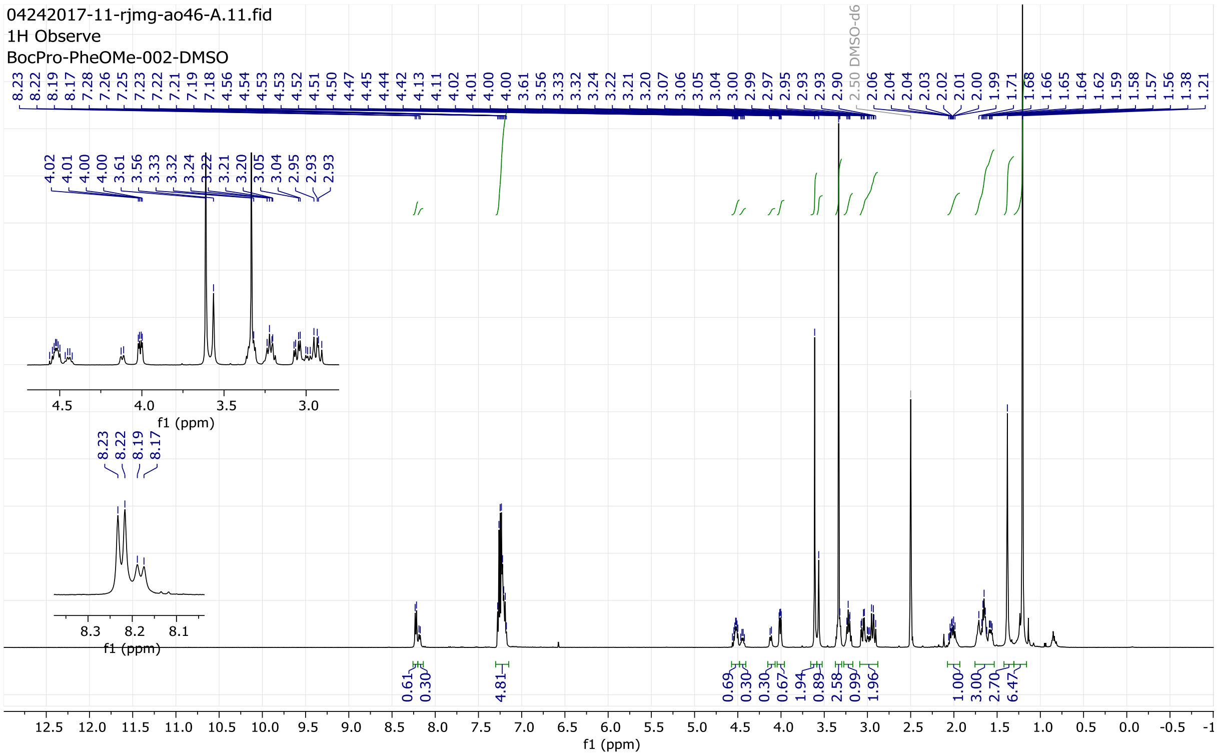

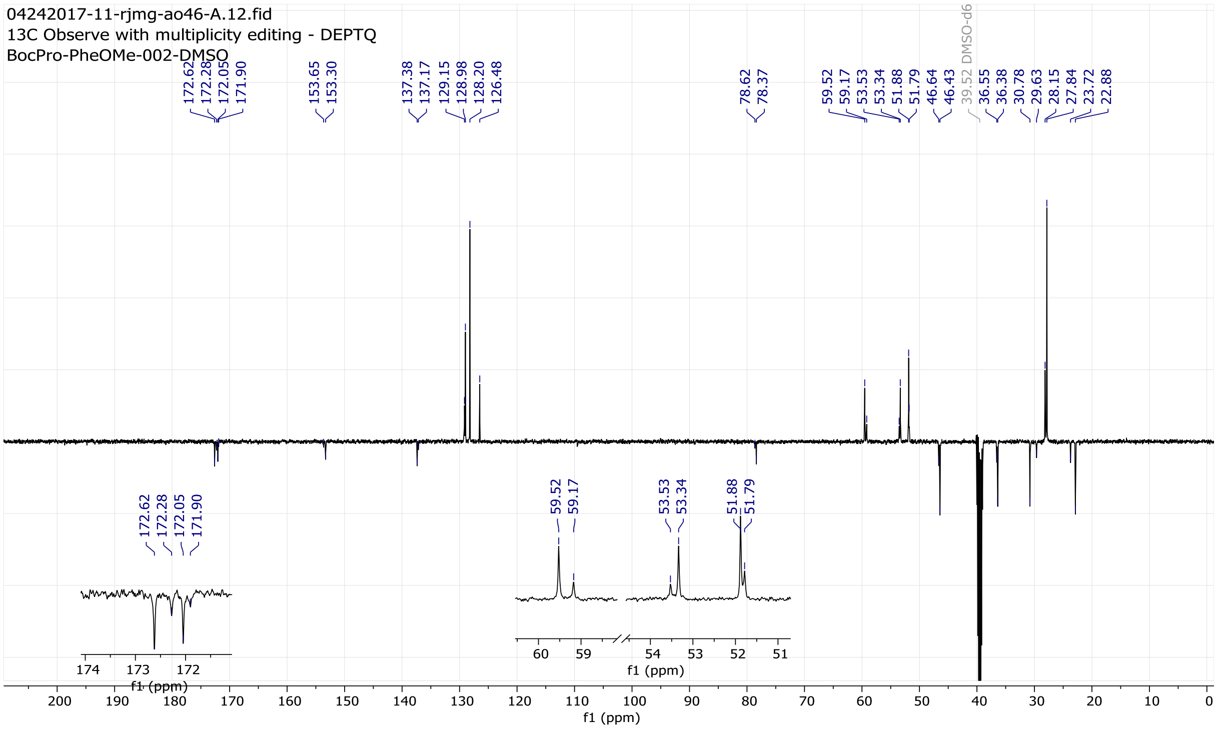

^{1}H and ^{13}C NMR of Boc-L-Pro-L-Phe-OMe (4)

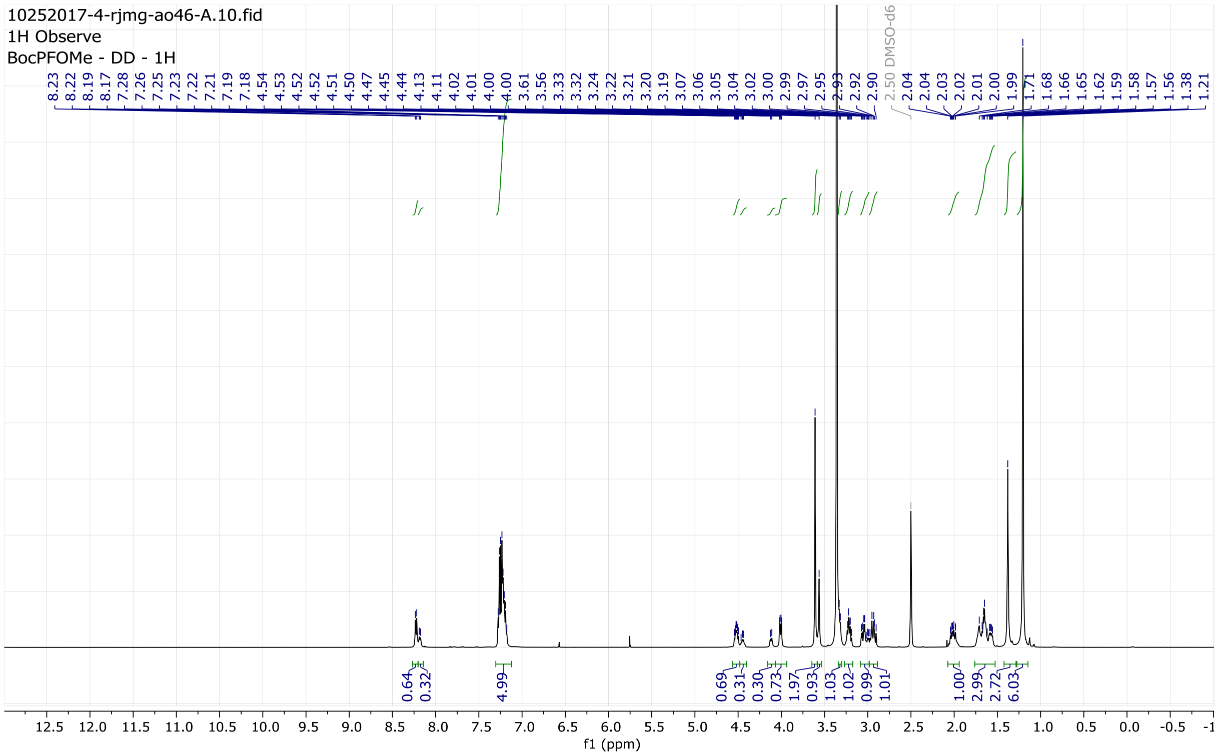

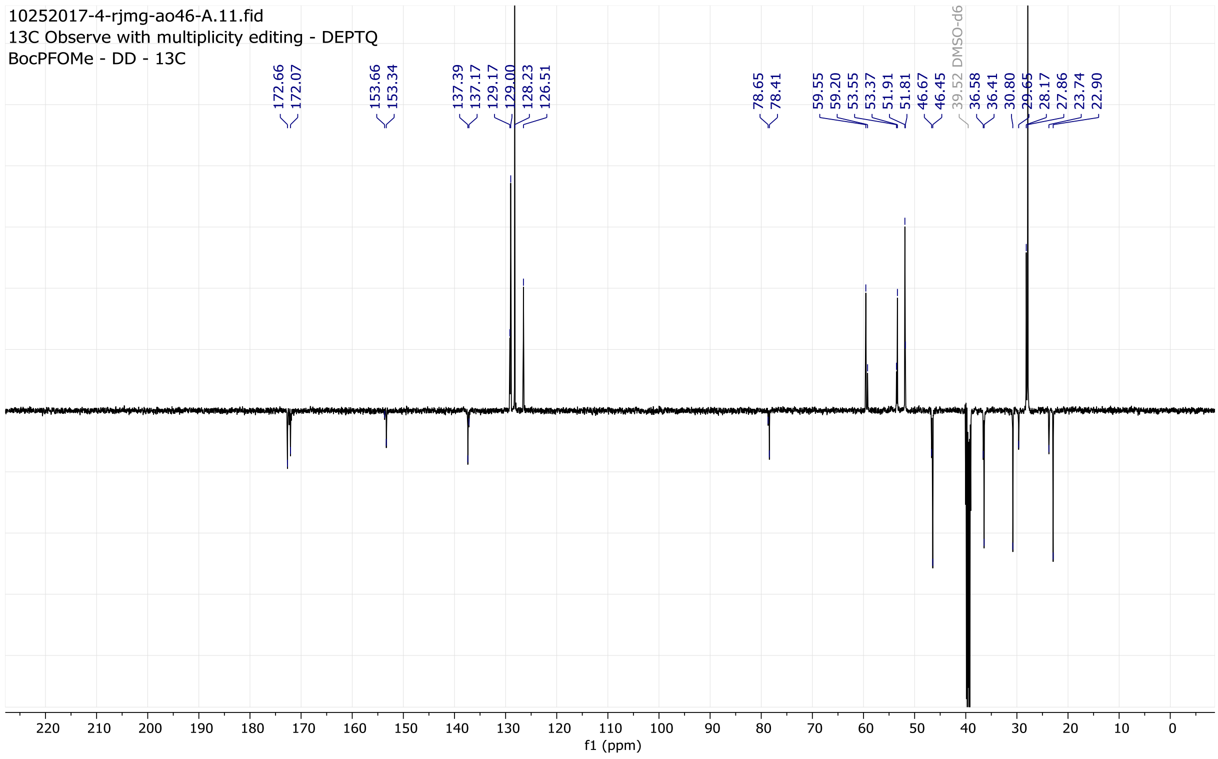

^{1}H and ^{13}C NMR of Boc-D-Pro-D-Phe-OMe

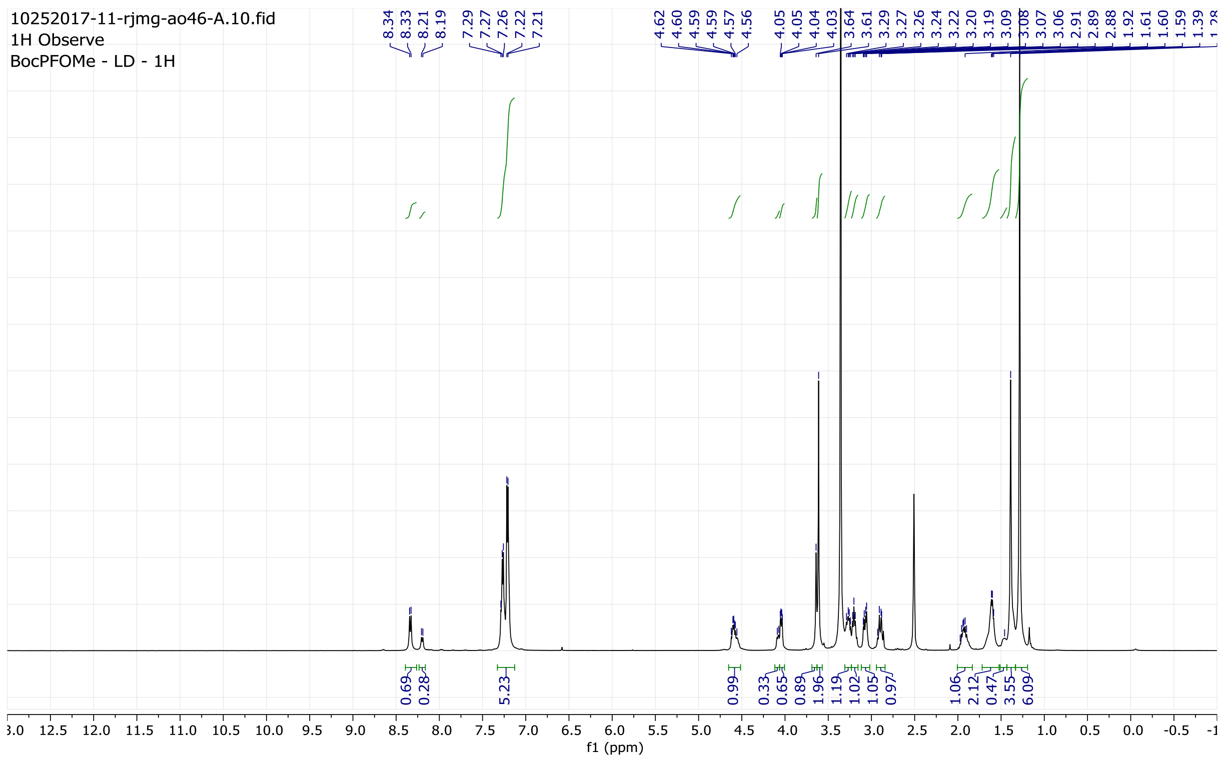

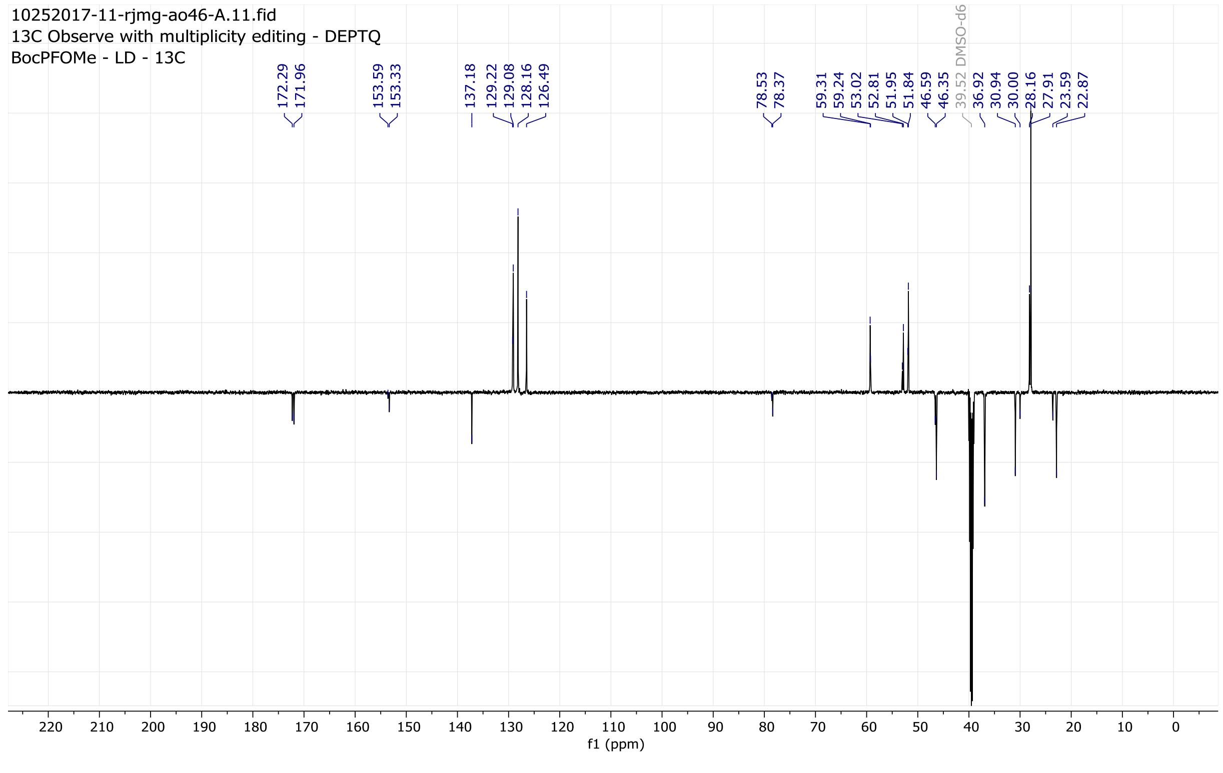

^{1}H and ^{13}C NMR of Boc-L-Pro-D-Phe-OMe

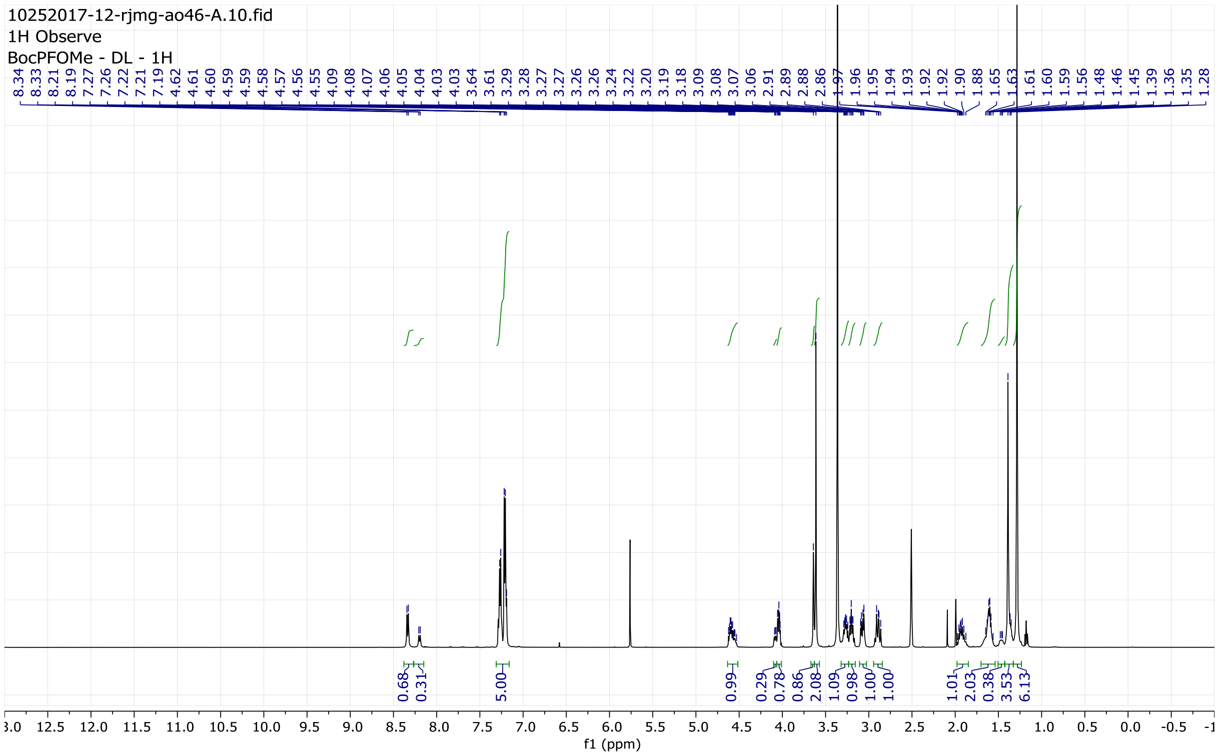

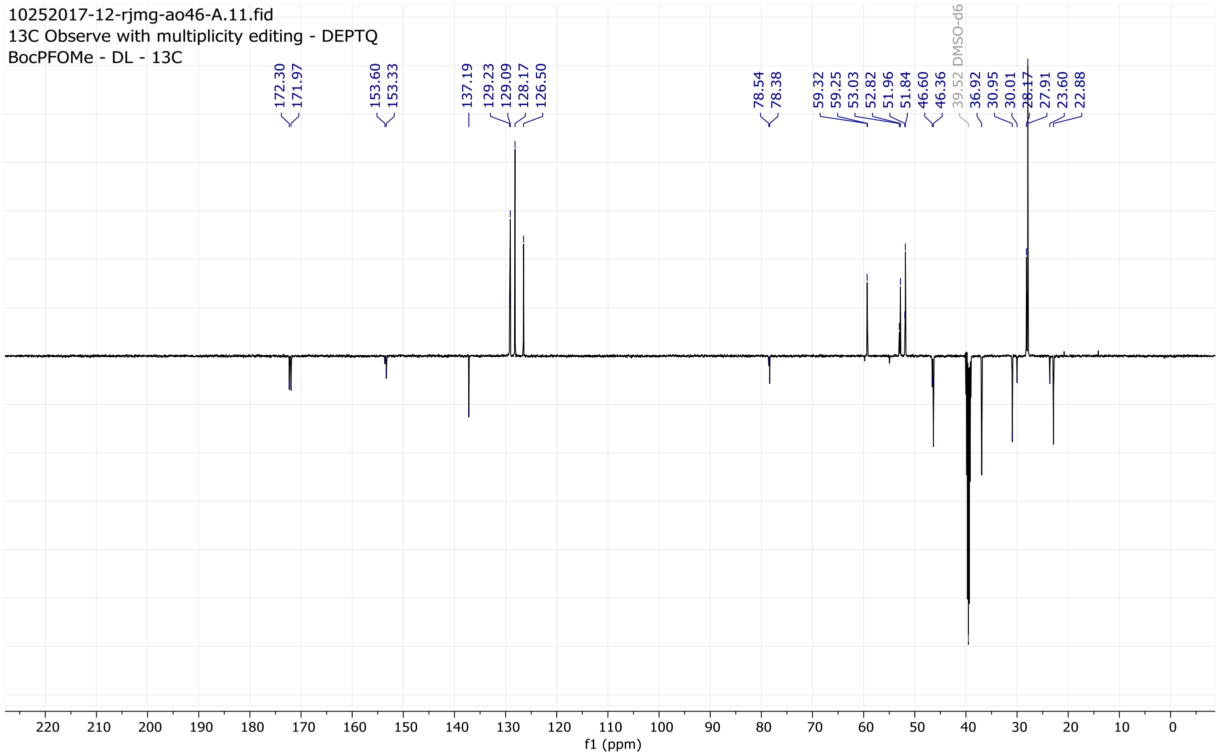

^{1}H and ^{13}C NMR of Boc-D-Pro-L-Phe-OMe

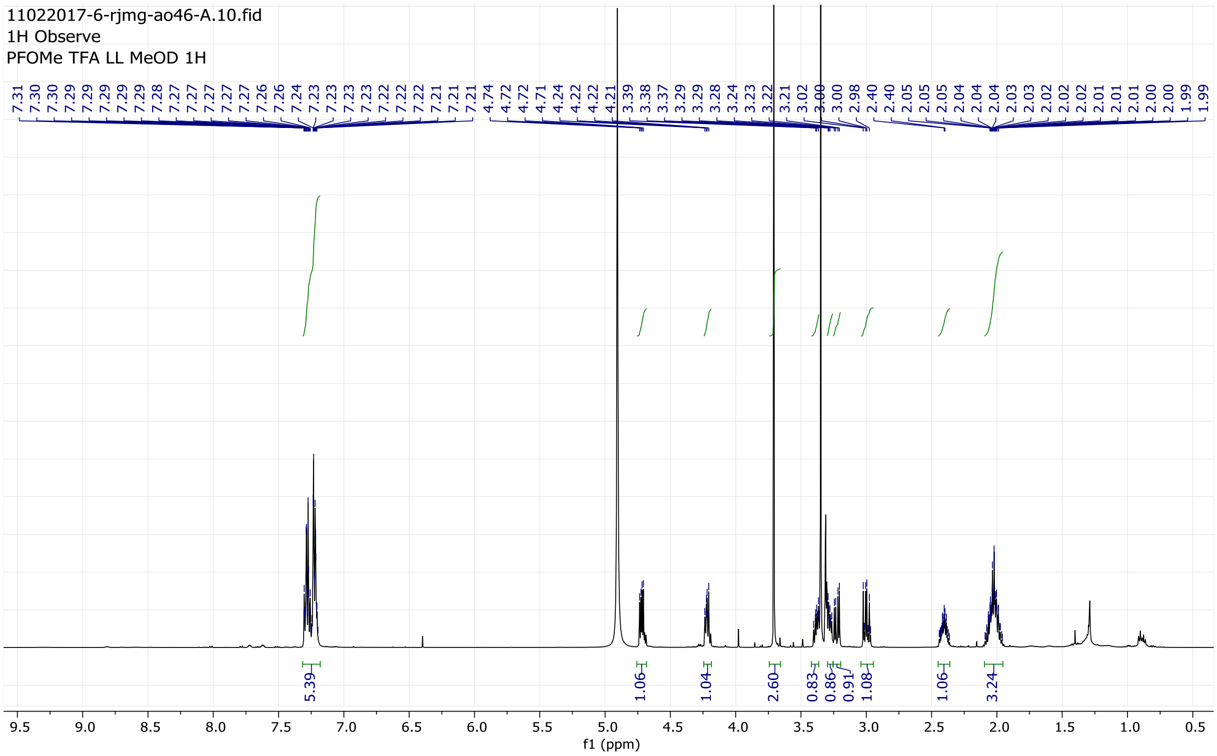

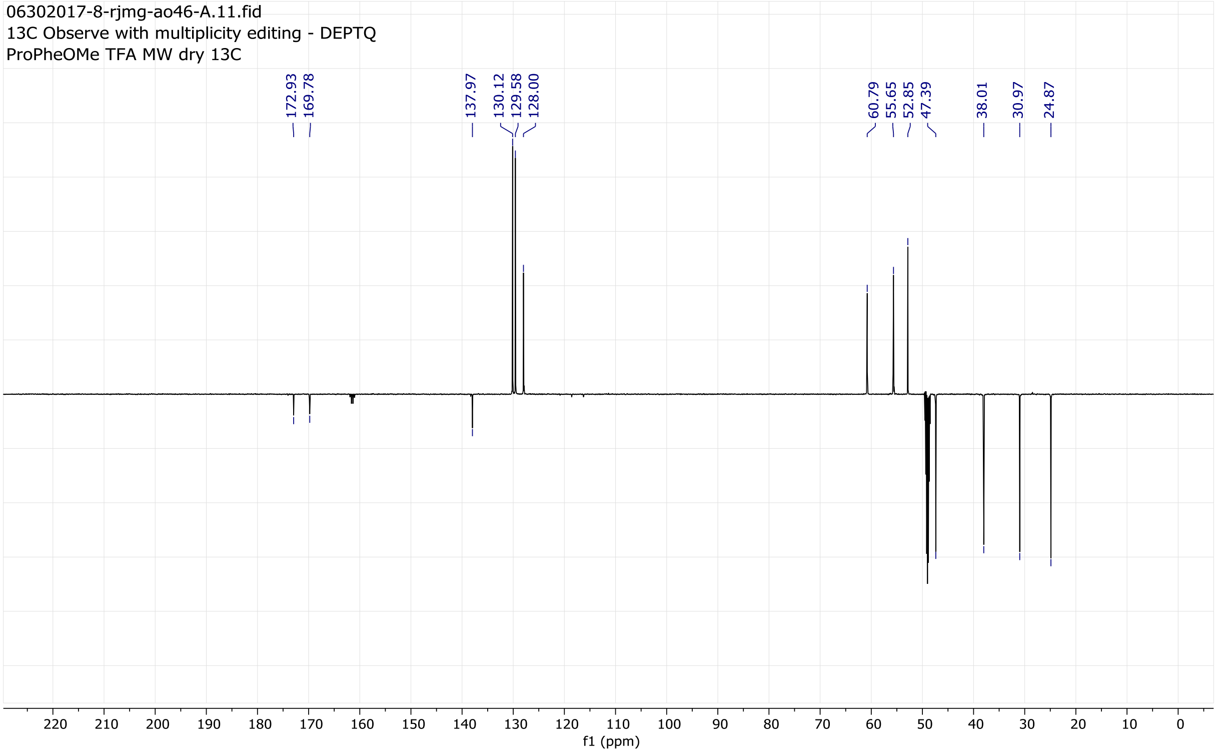

^{1}H and ^{13}C NMR of L-Pro-L-Phe-OMe.TFA (5)

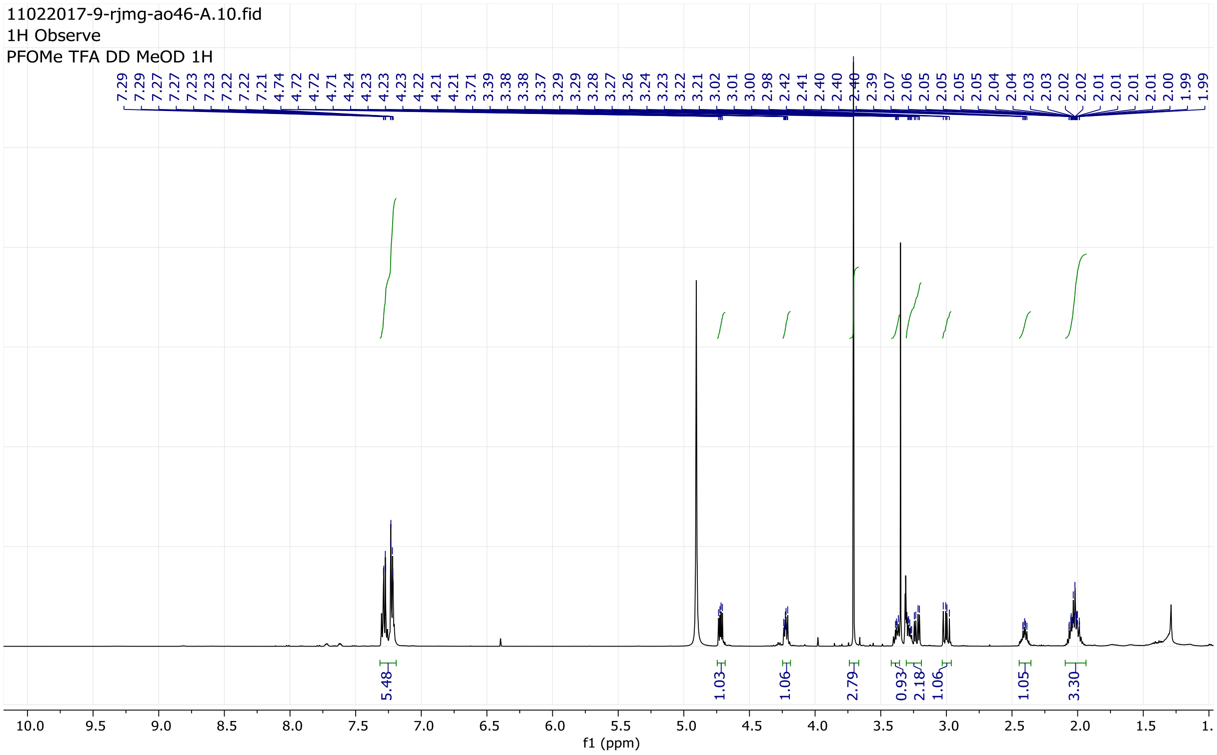

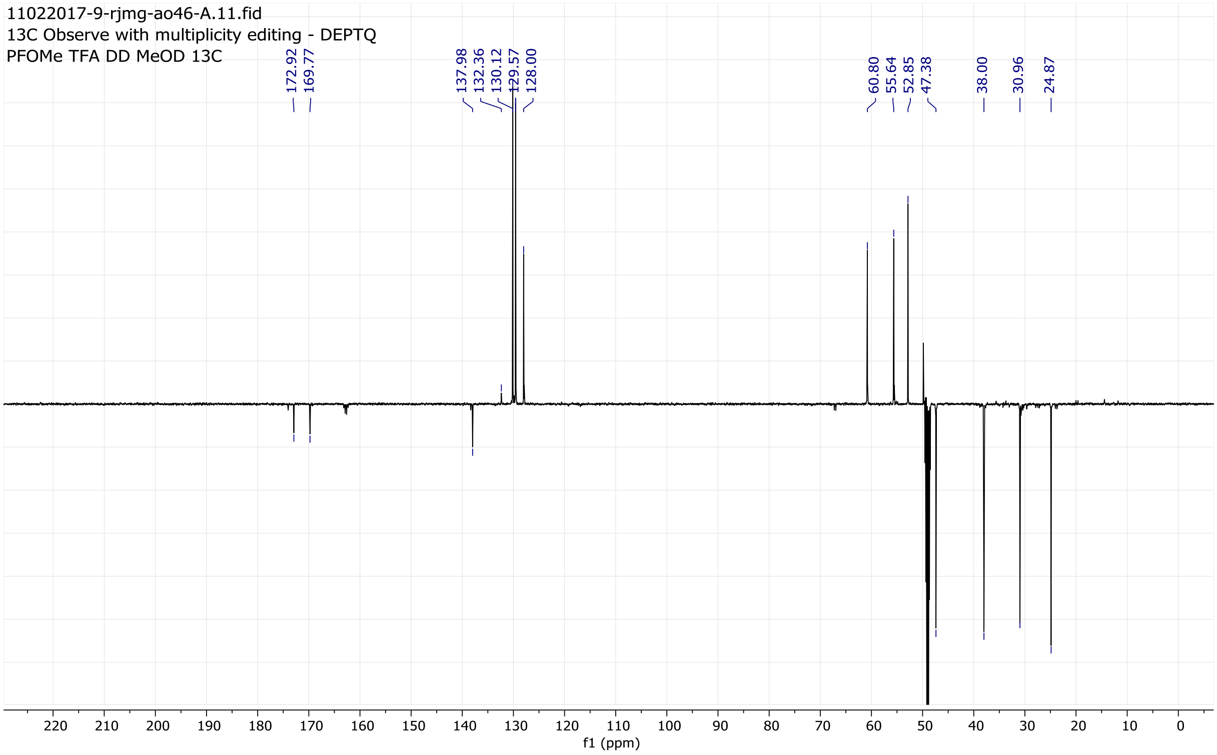

^{1}H and ^{13}C NMR of D-Pro-D-Phe-OMe.TFA

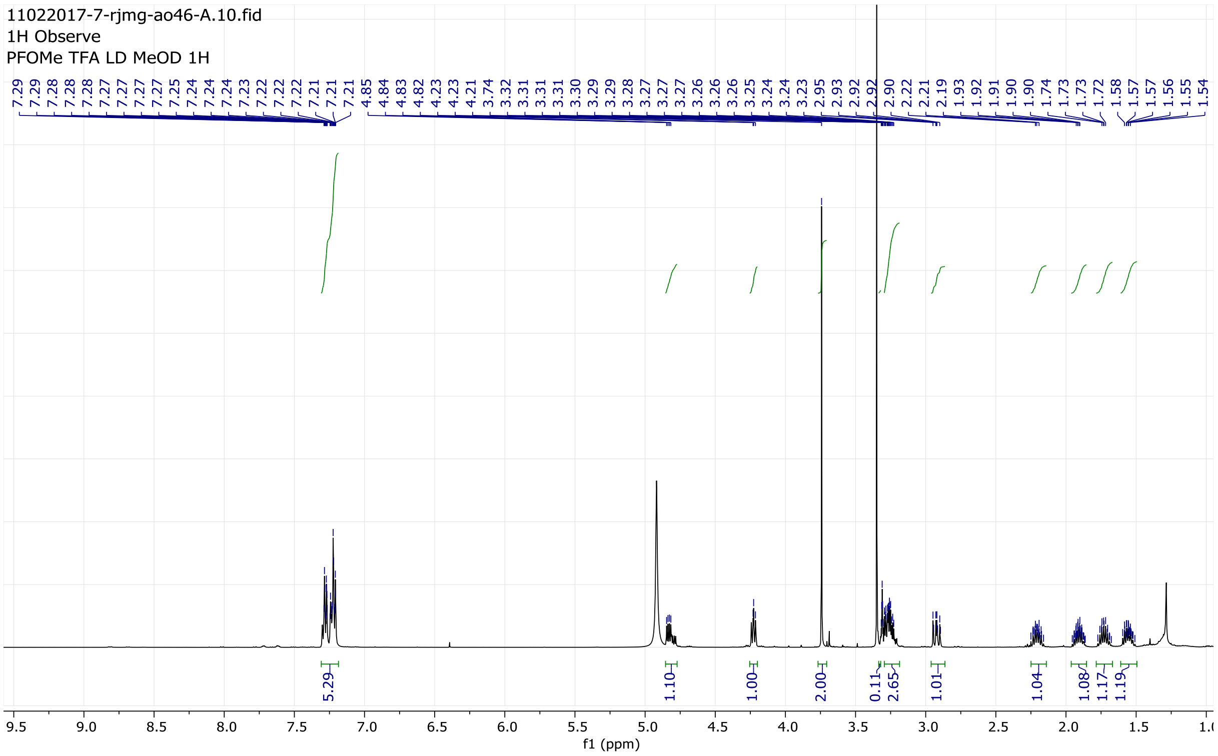

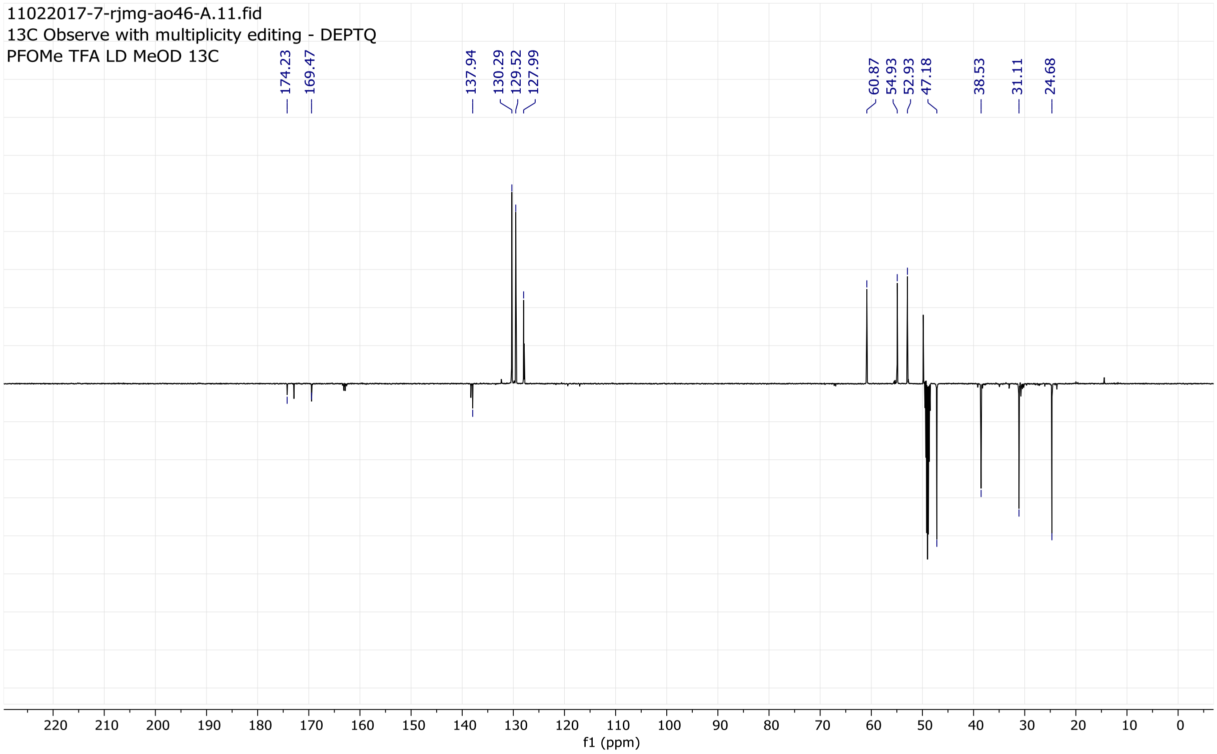

^{1}H and ^{13}C NMR of L-Pro-D-Phe-OMe.TFA

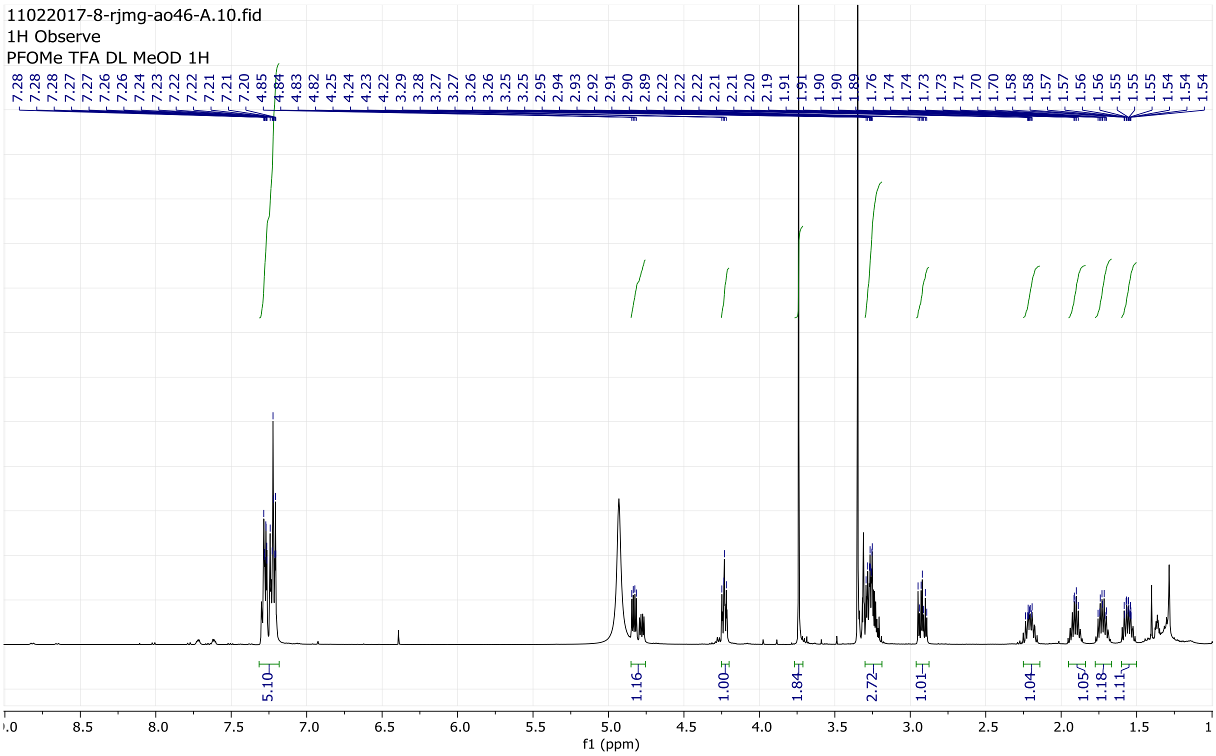

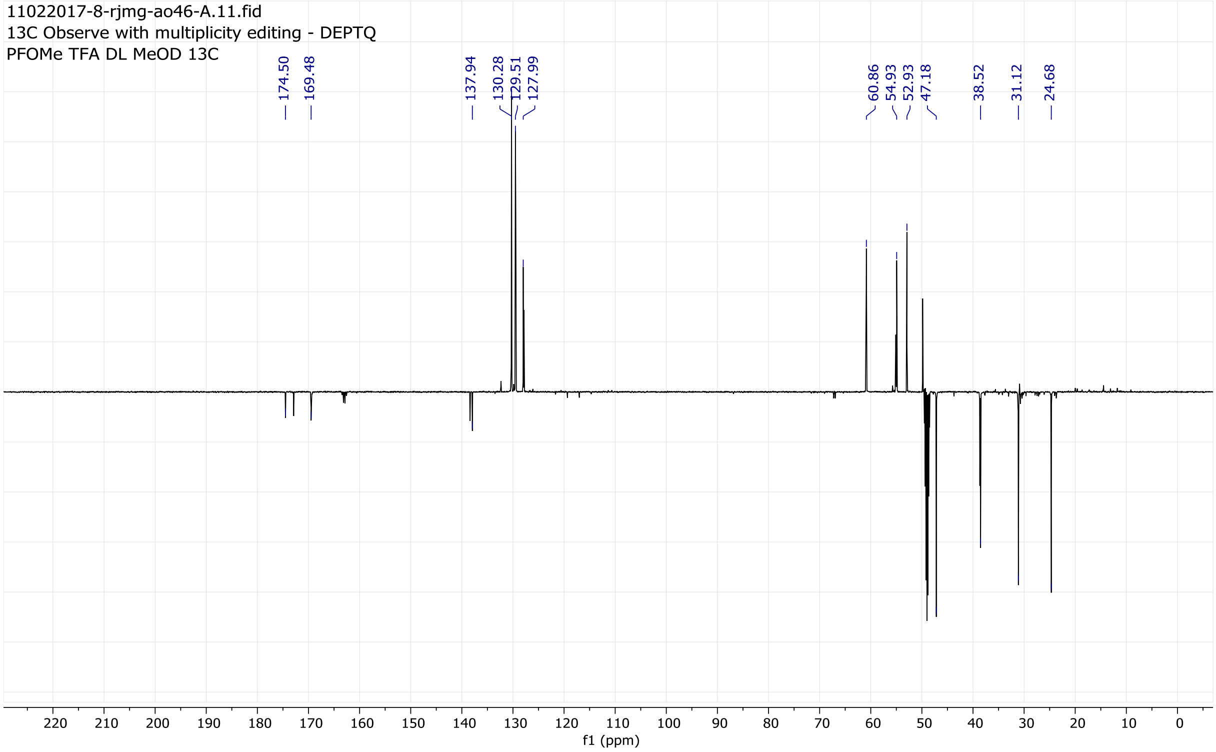

^{1}H and ^{13}C NMR of D-Pro-L-Phe-OMe.TFA

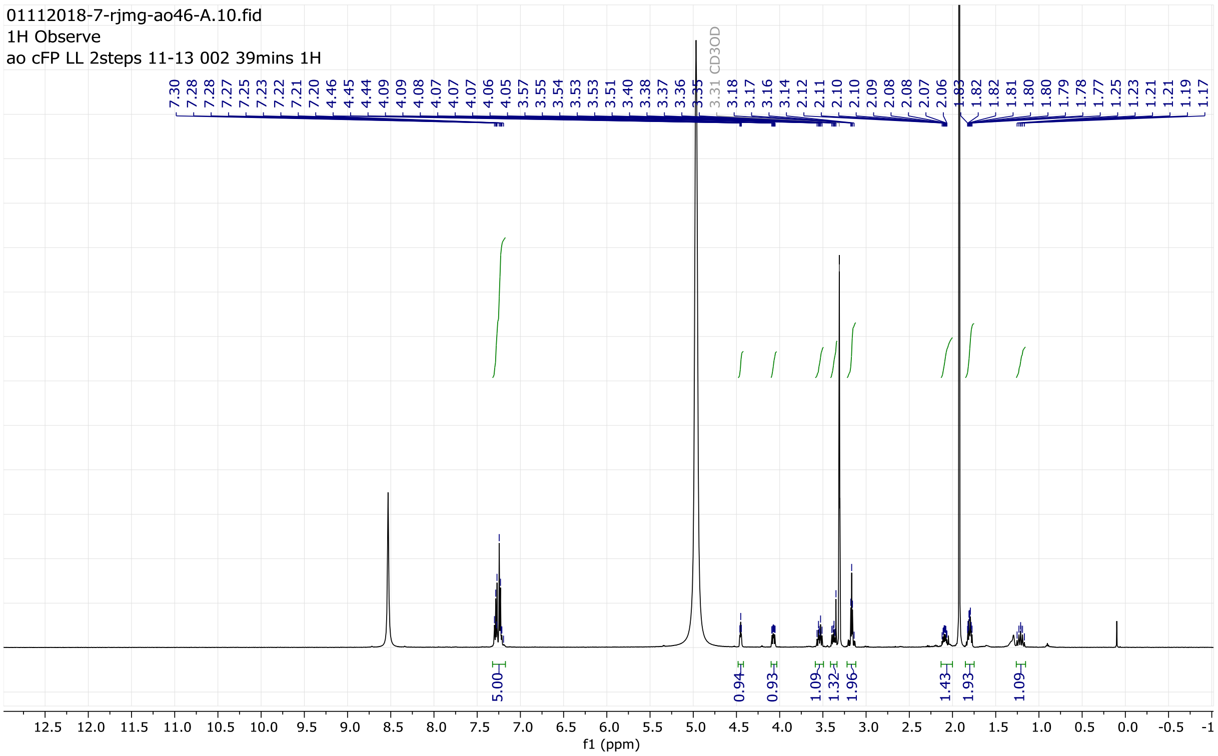

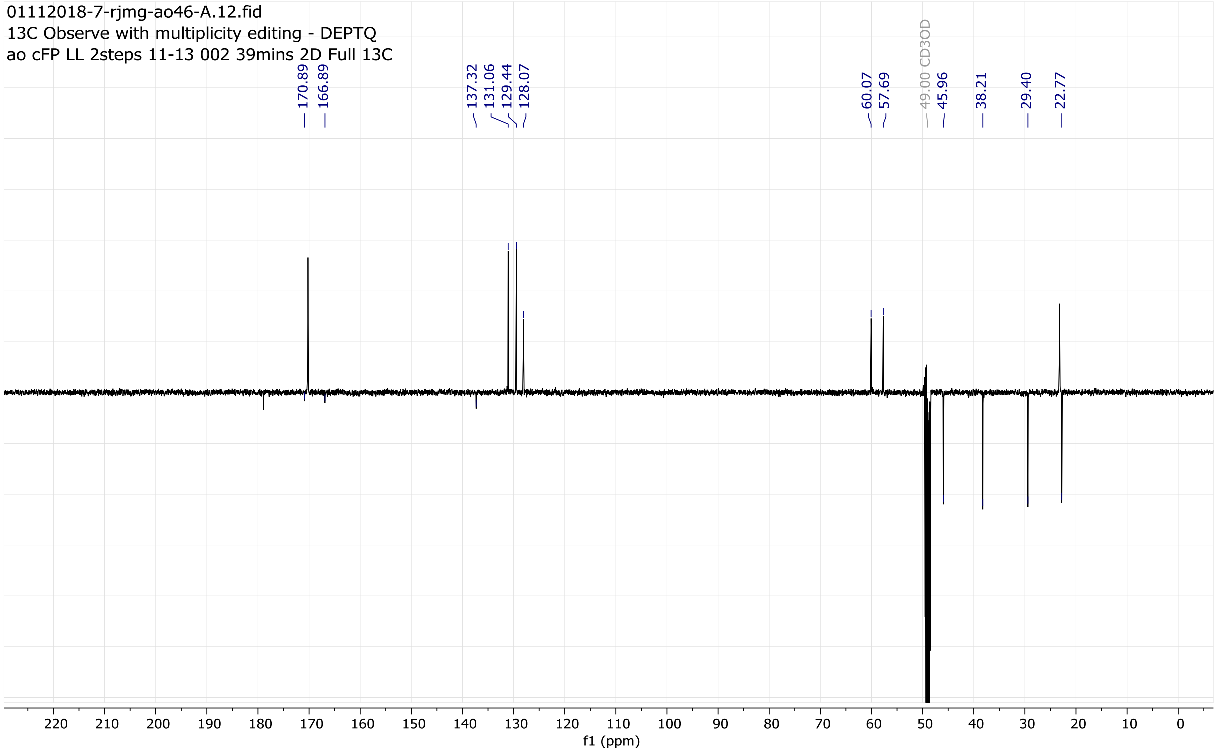

^{1}H and ^{13}C NMR of cyclo-(L-Phe-L-Pro)

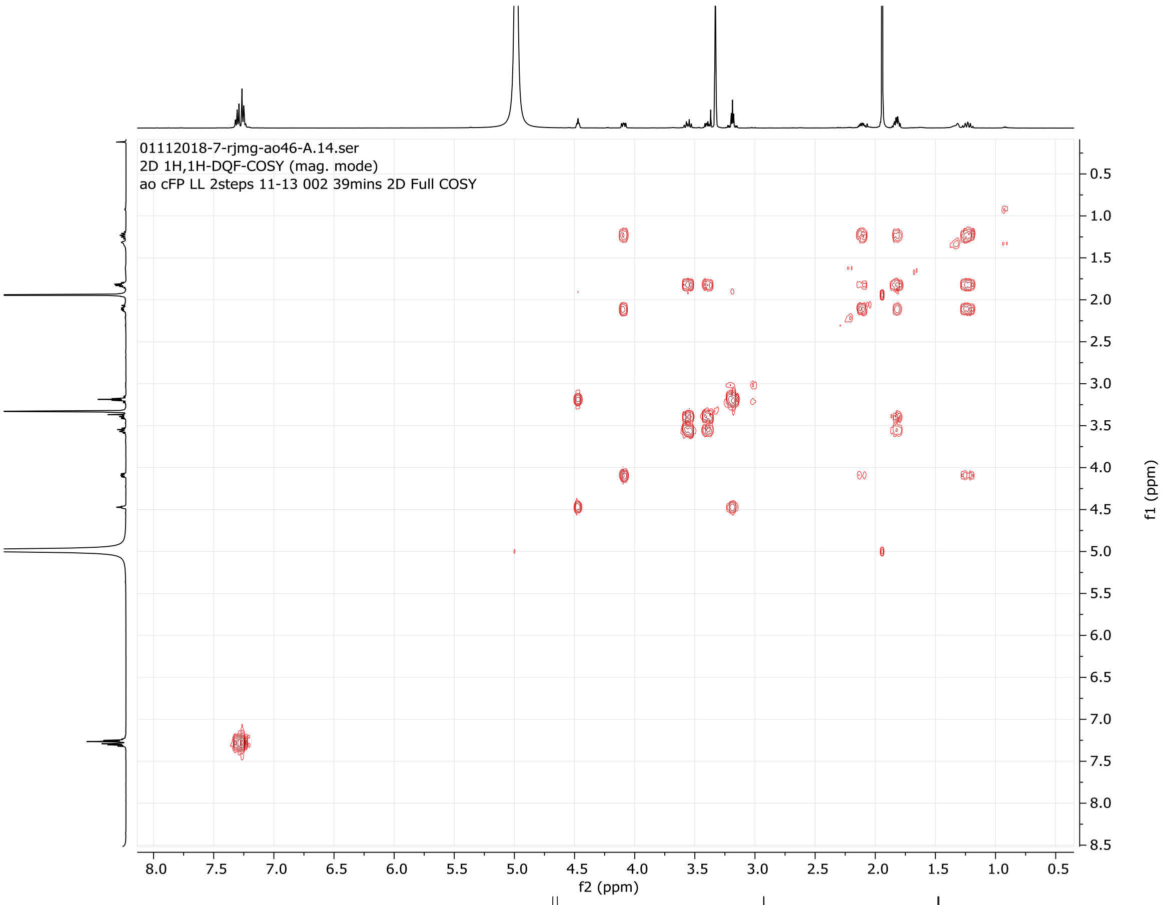

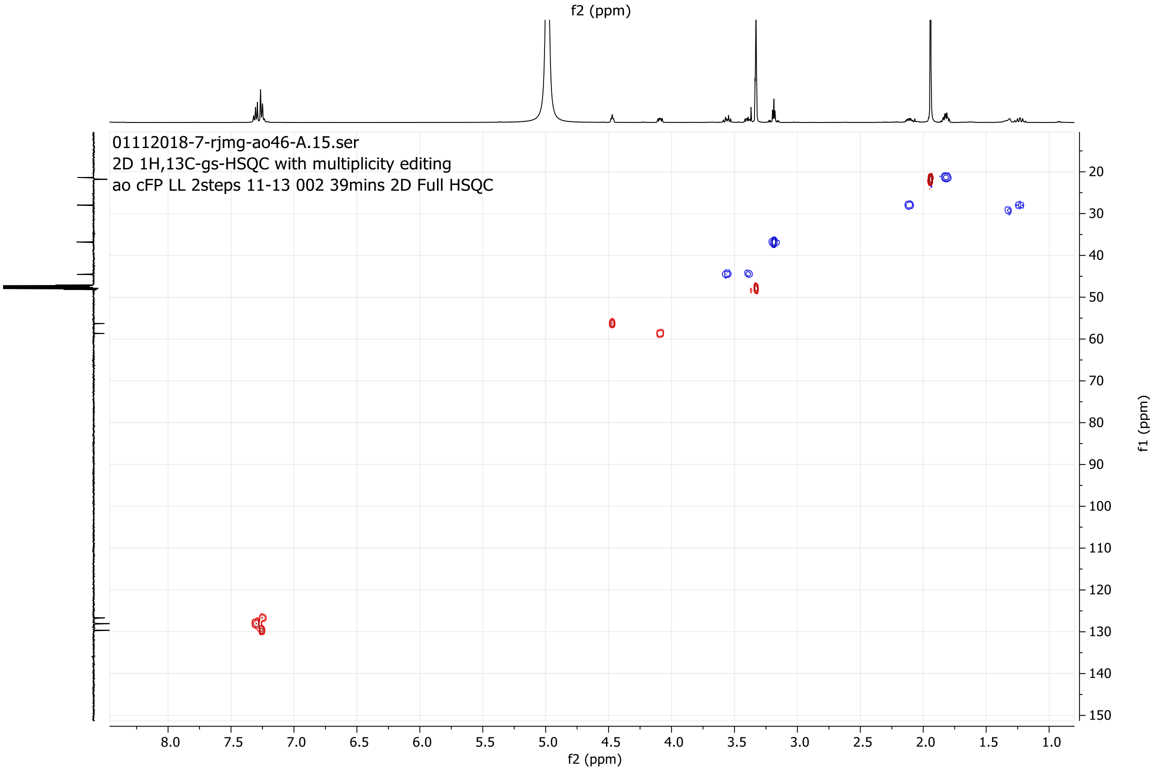

COSY and HSQC NMR of cyclo-(L-Phe-L-Pro)

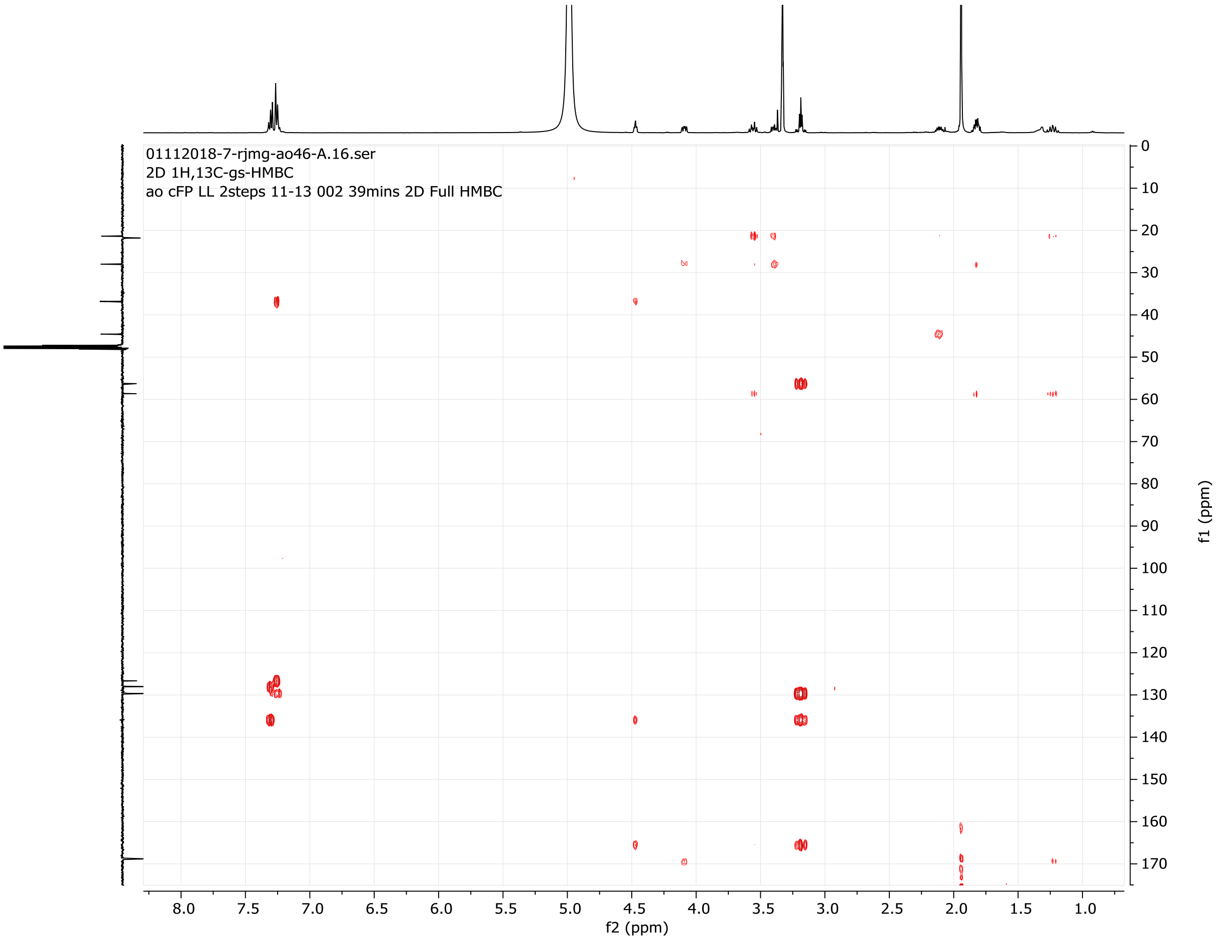

HMBC NMR of cyclo-(L-Phe-L-Pro)

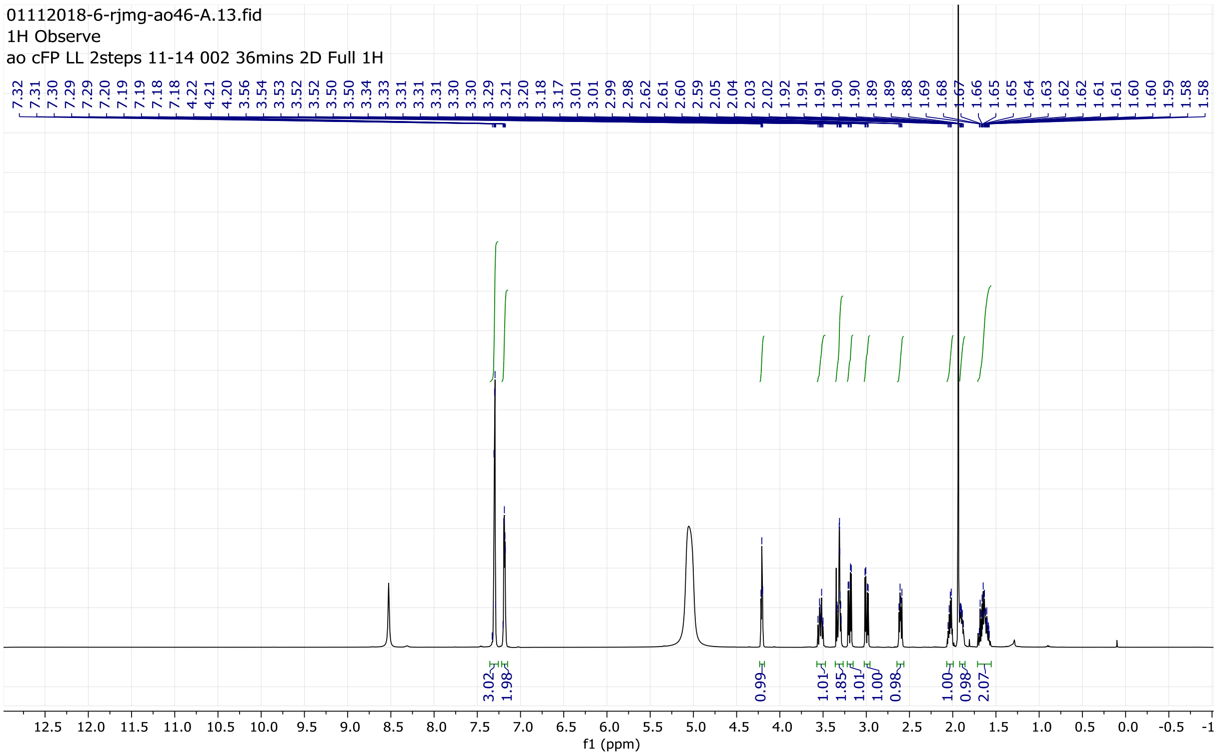

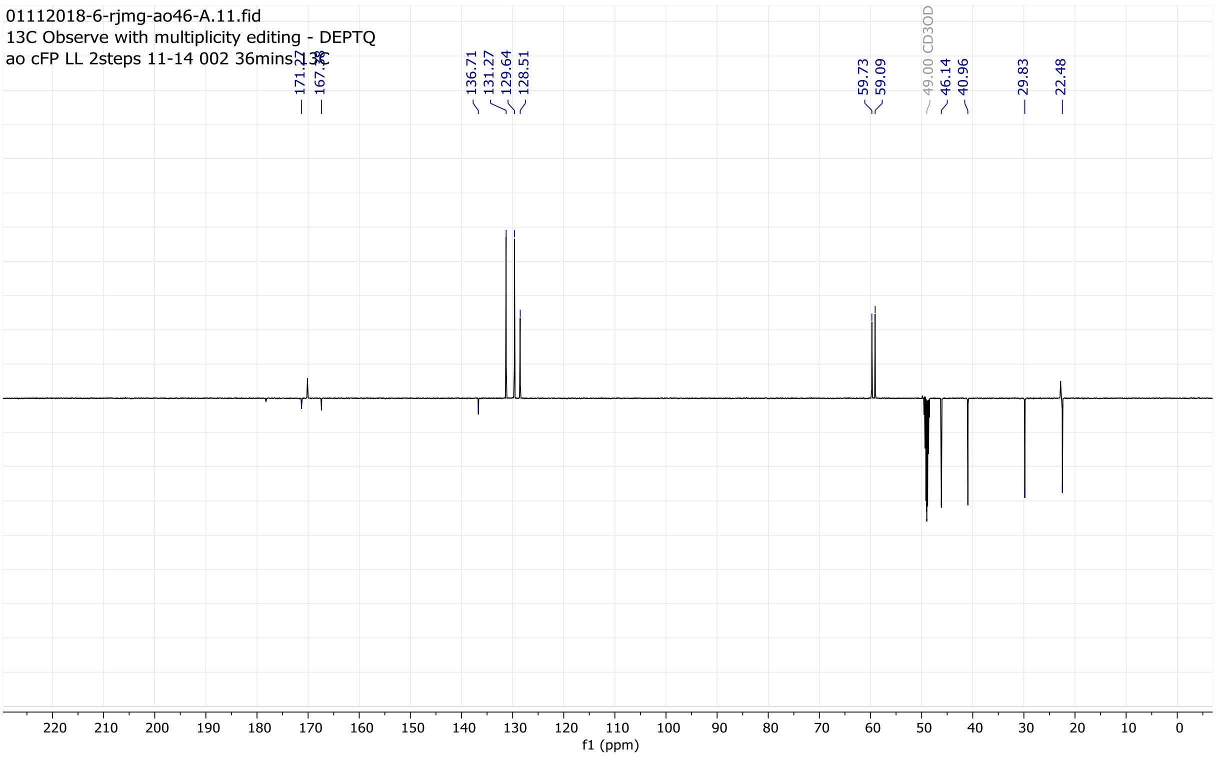

^{1}H and ^{13}C NMR of cyclo-(L-Phe-D-Pro)

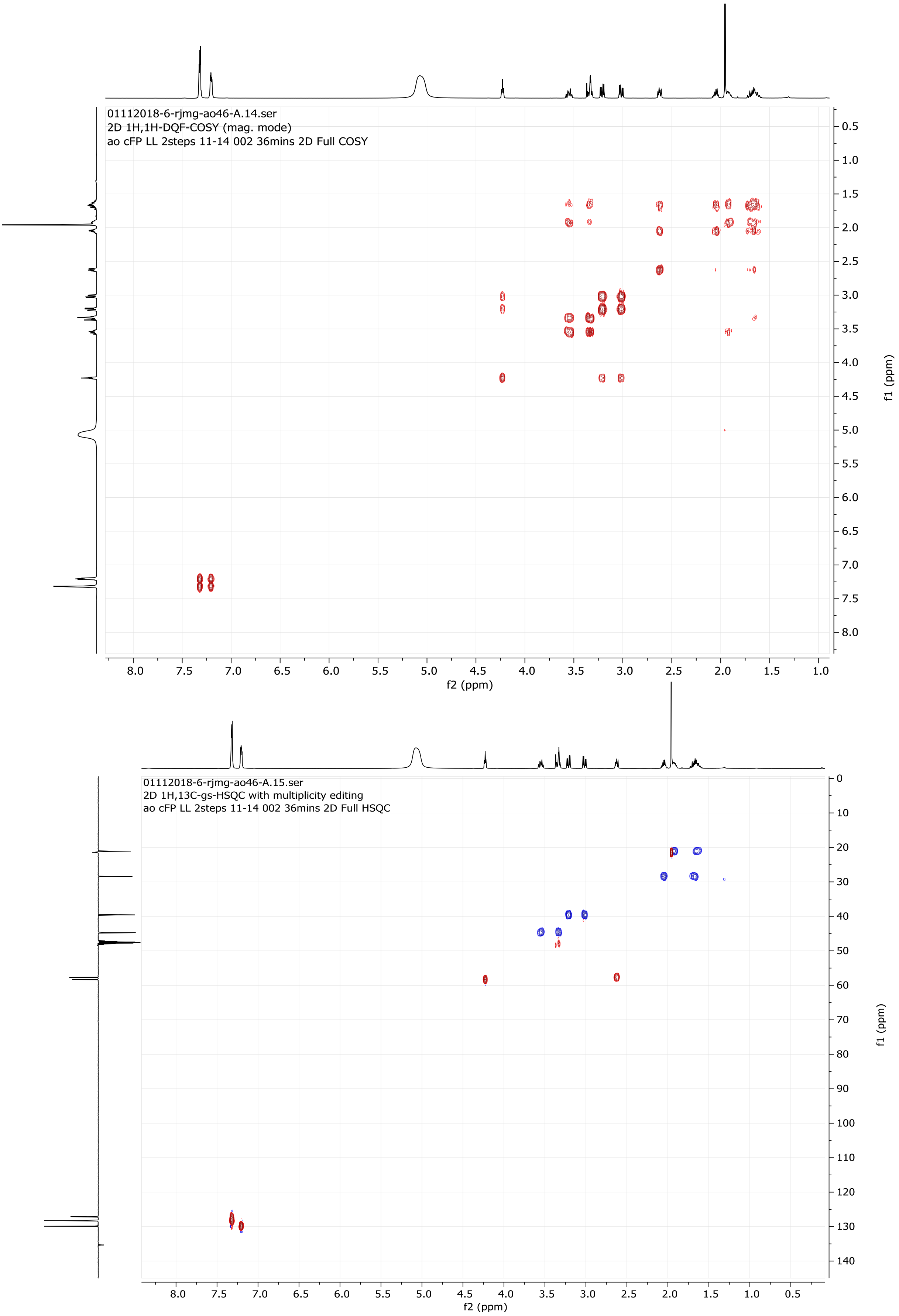

COSY and HSQC NMR of cyclo-(L-Phe-D-Pro)

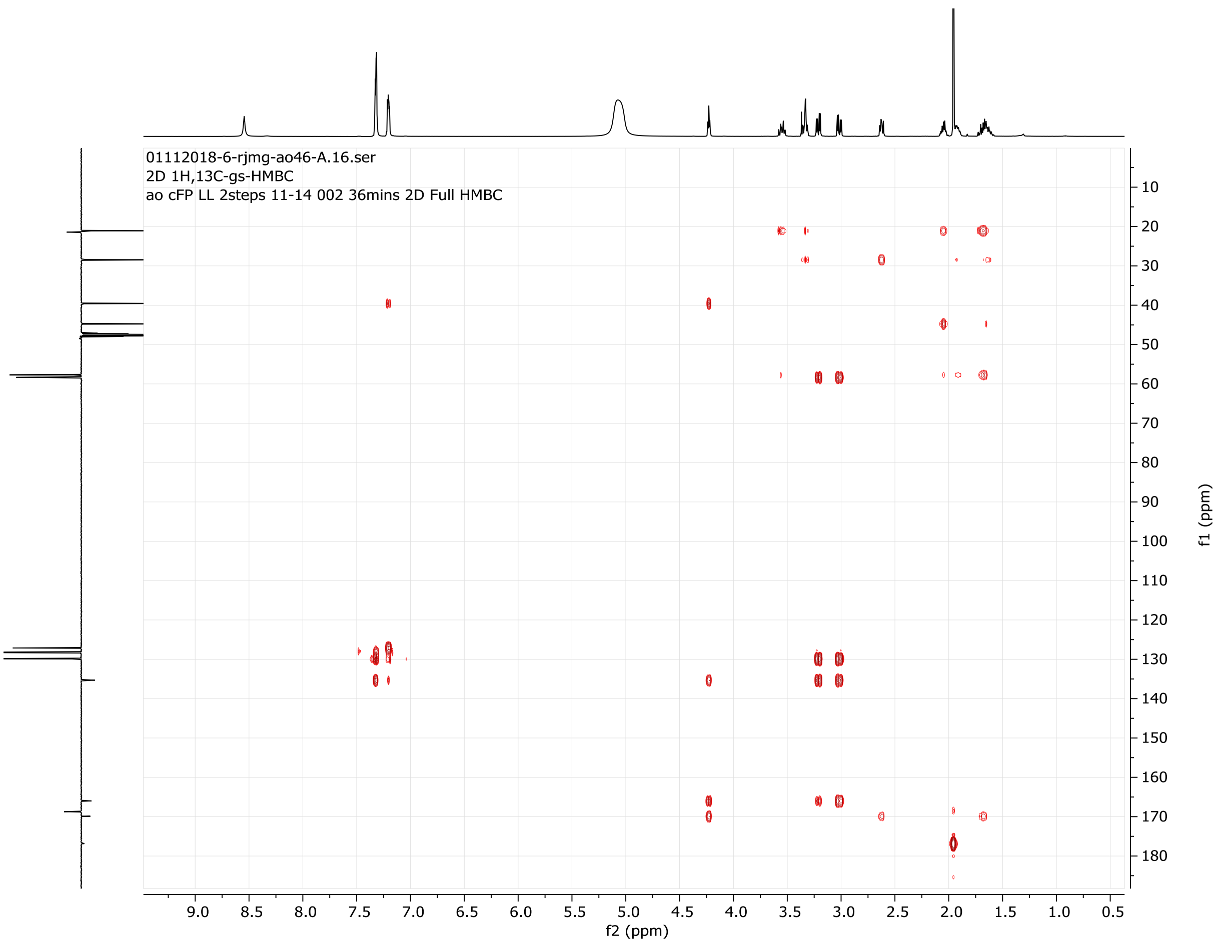

HMBC NMR of cyclo-(L-Phe-D-Pro)

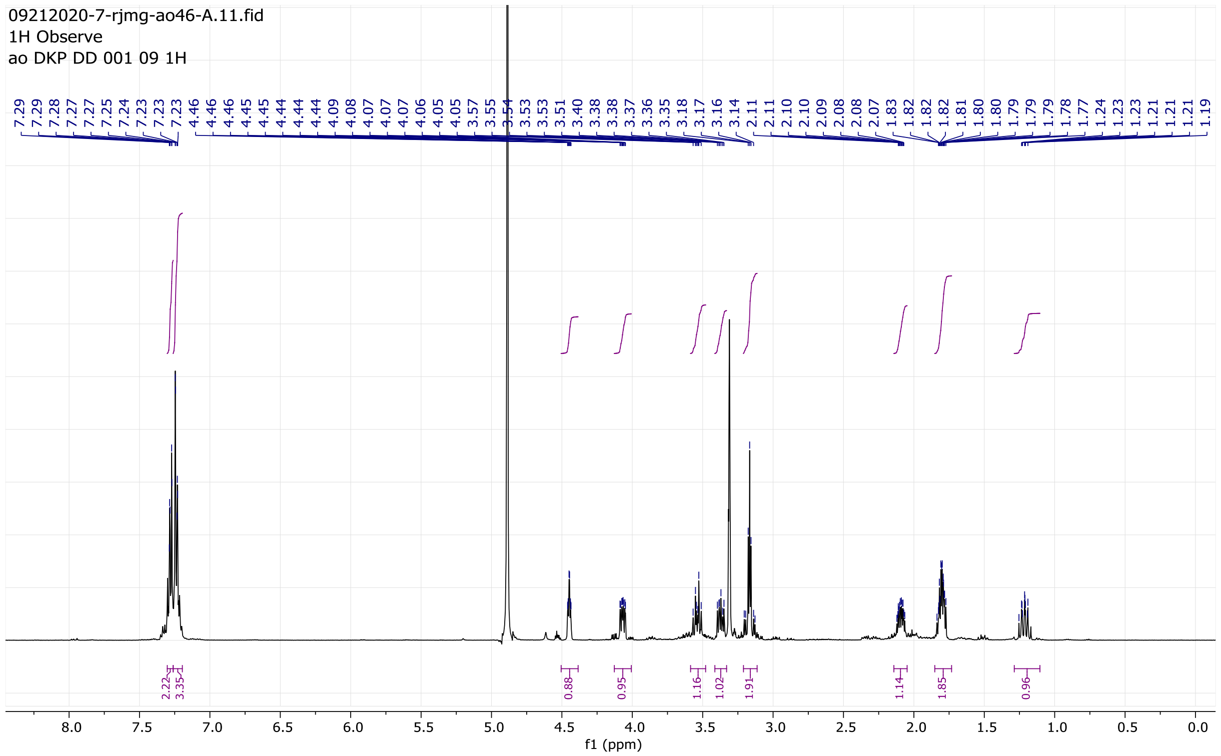

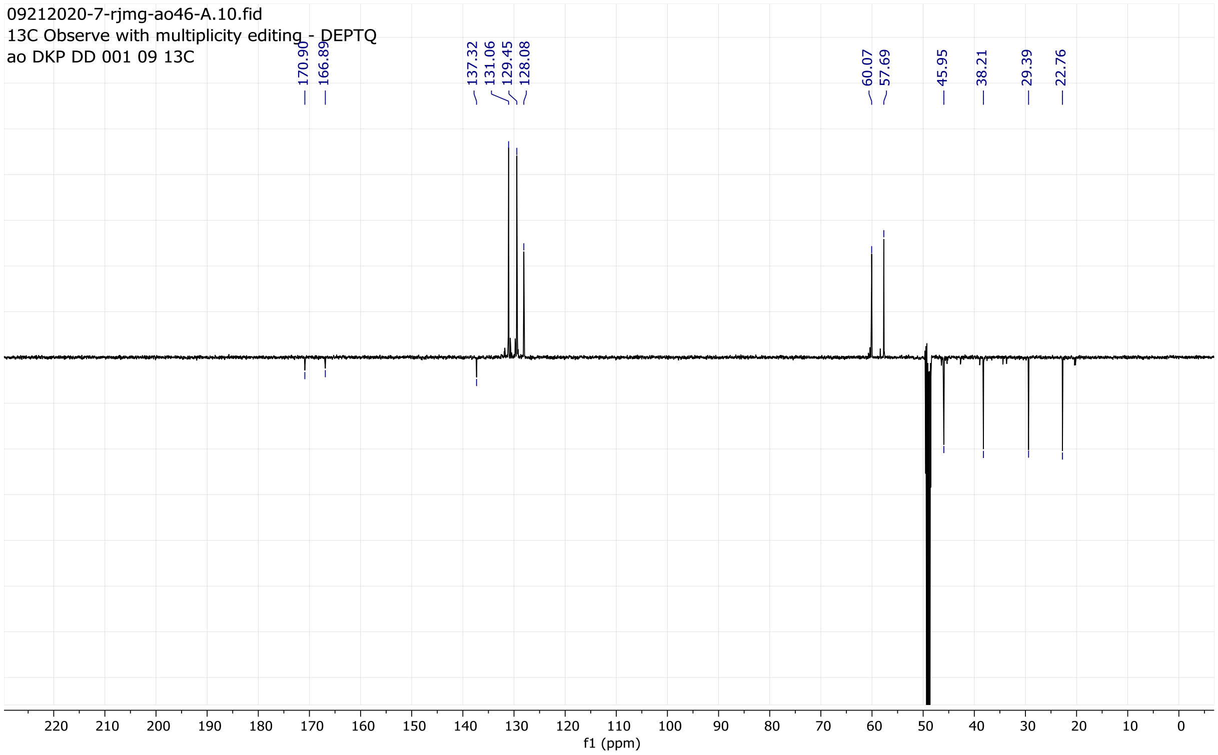

^{1}H and ^{13}C NMR of cyclo-(D-Phe-D-Pro)

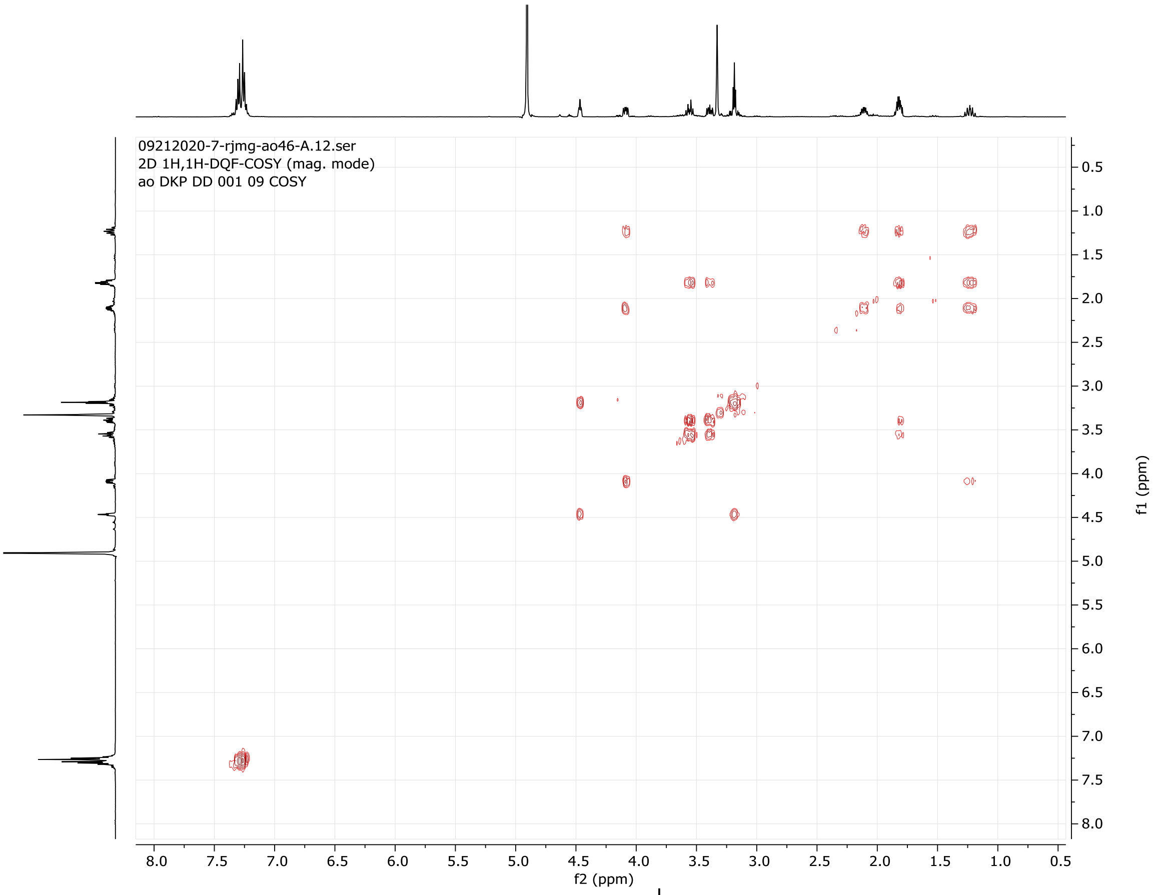

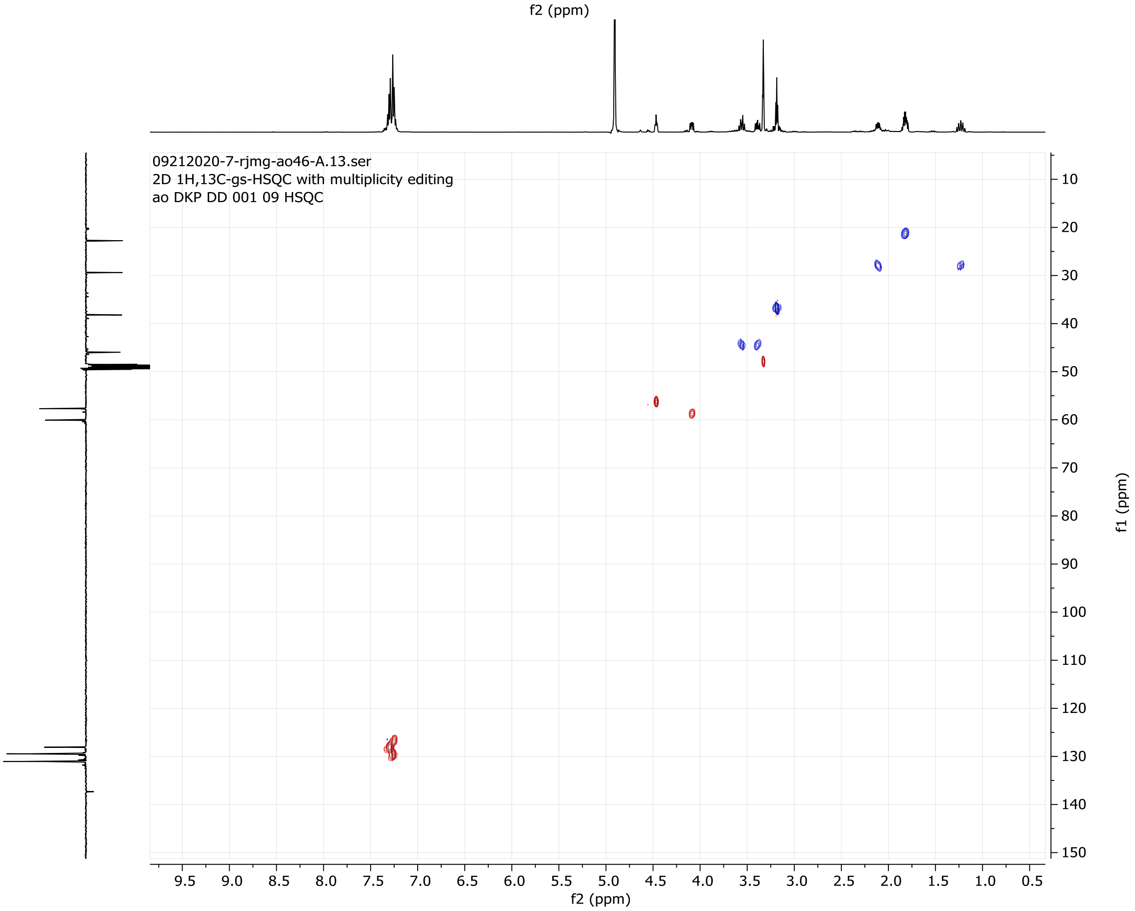

COSY and HSQC NMR of cyclo-(D-Phe-D-Pro)

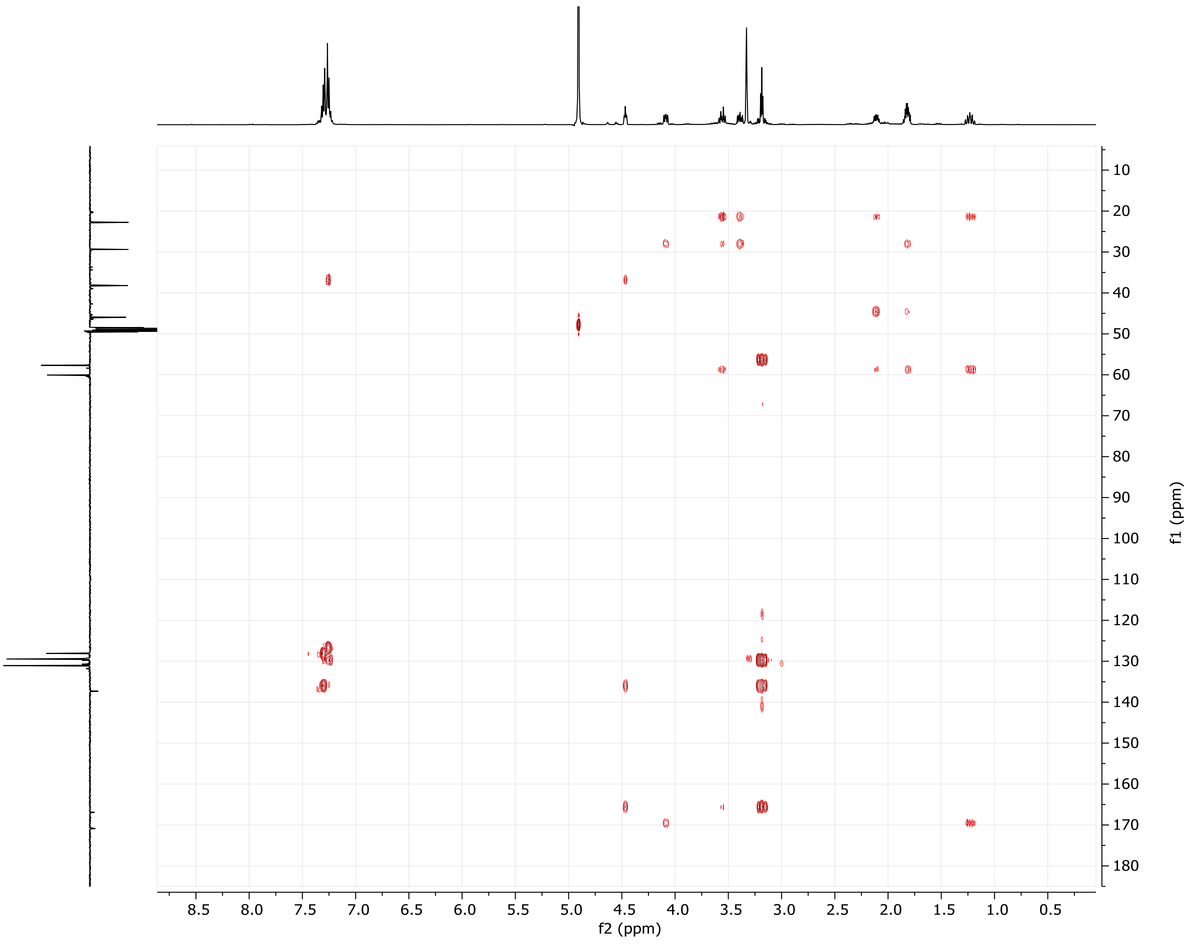

HMBC NMR of cyclo-(D-Phe-D-Pro)

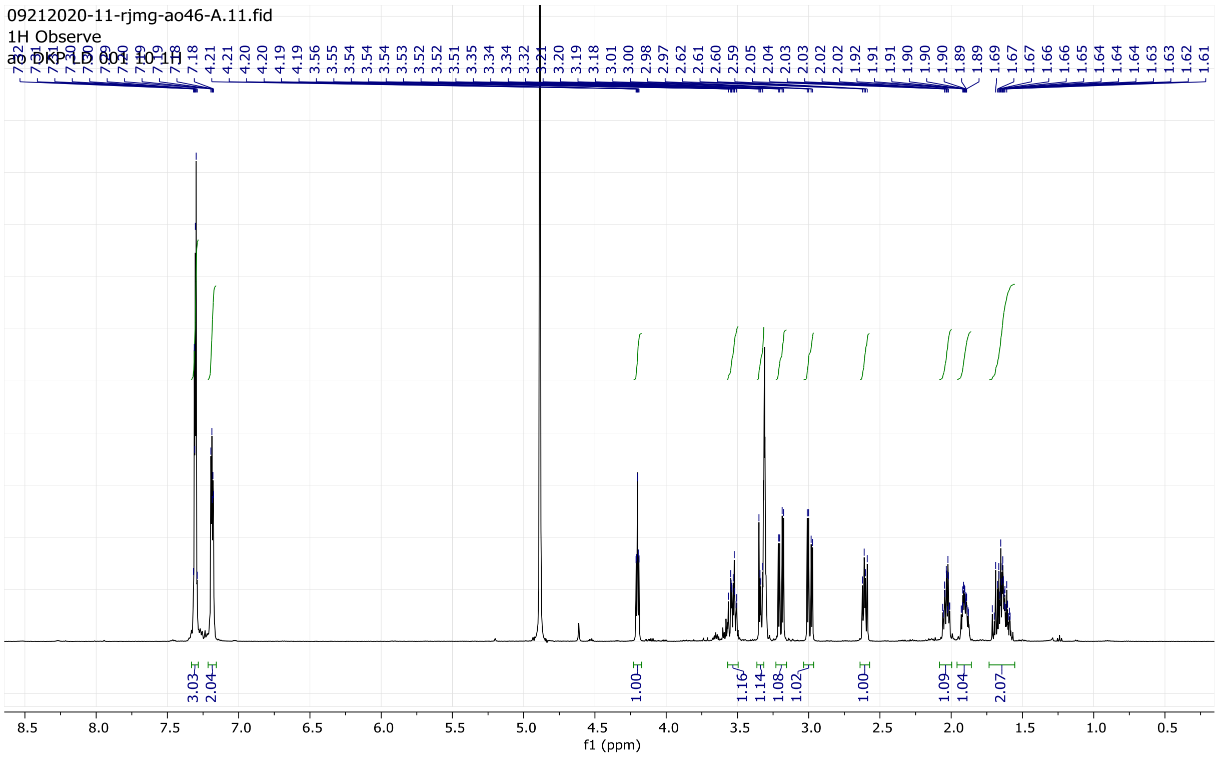

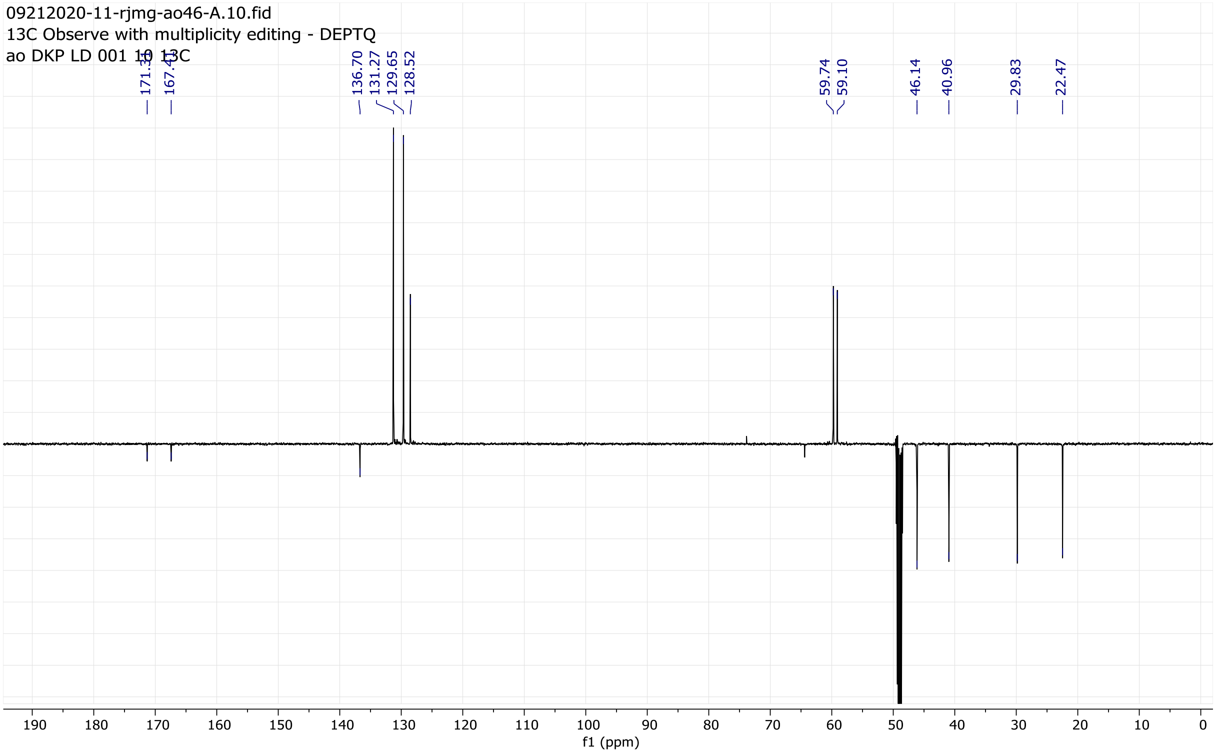

^{1}H and ^{13}C NMR of cyclo-(D-Phe-L-Pro)

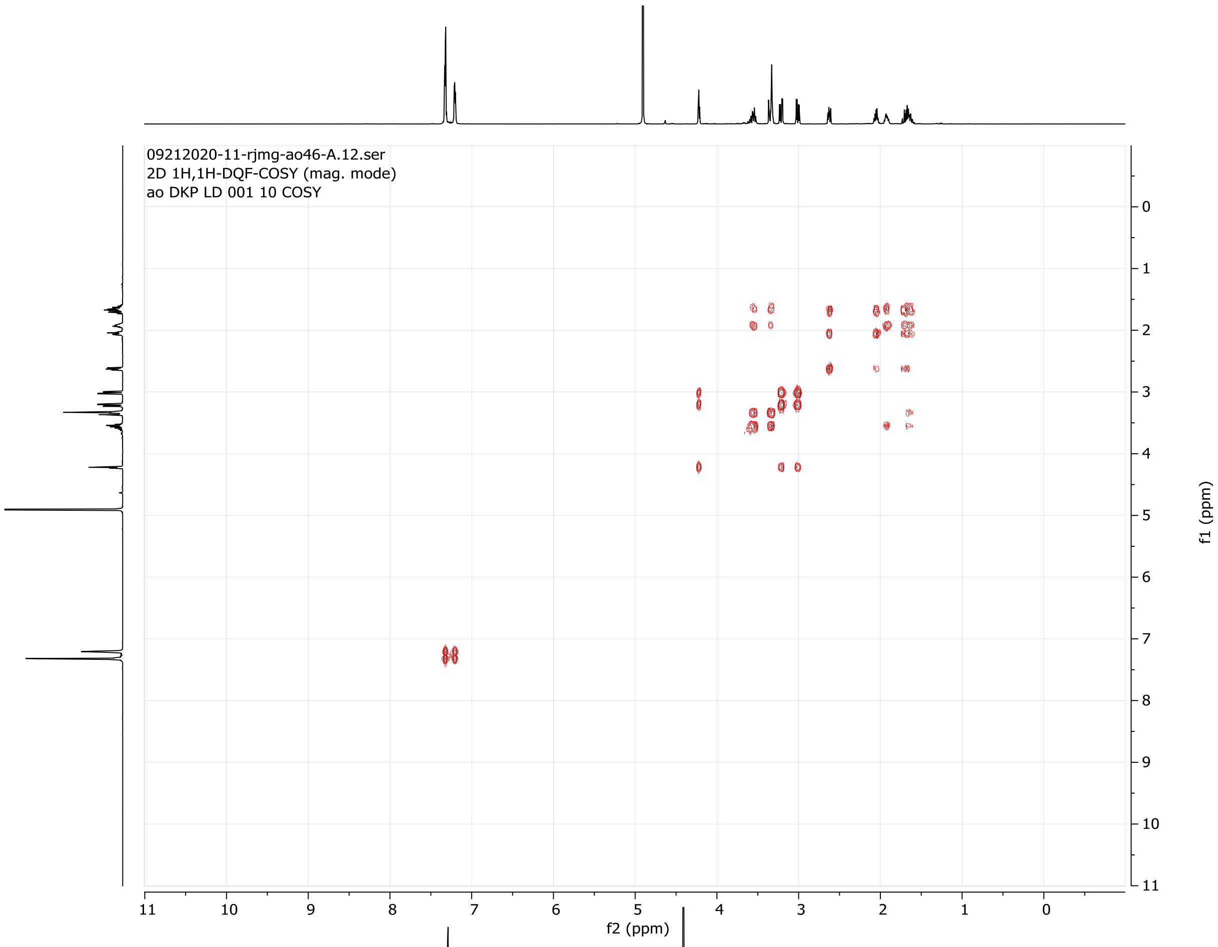

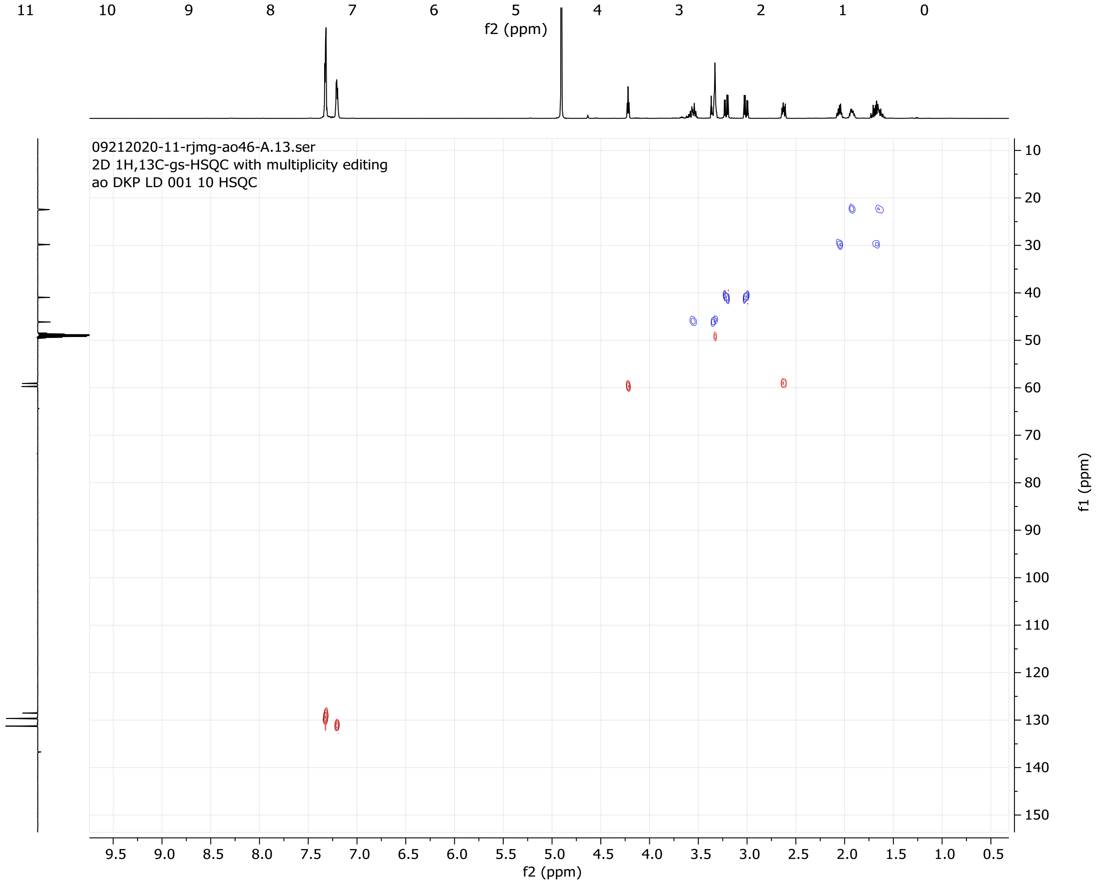

COSY and HSQC NMR of cyclo-(D-Phe-L-Pro)

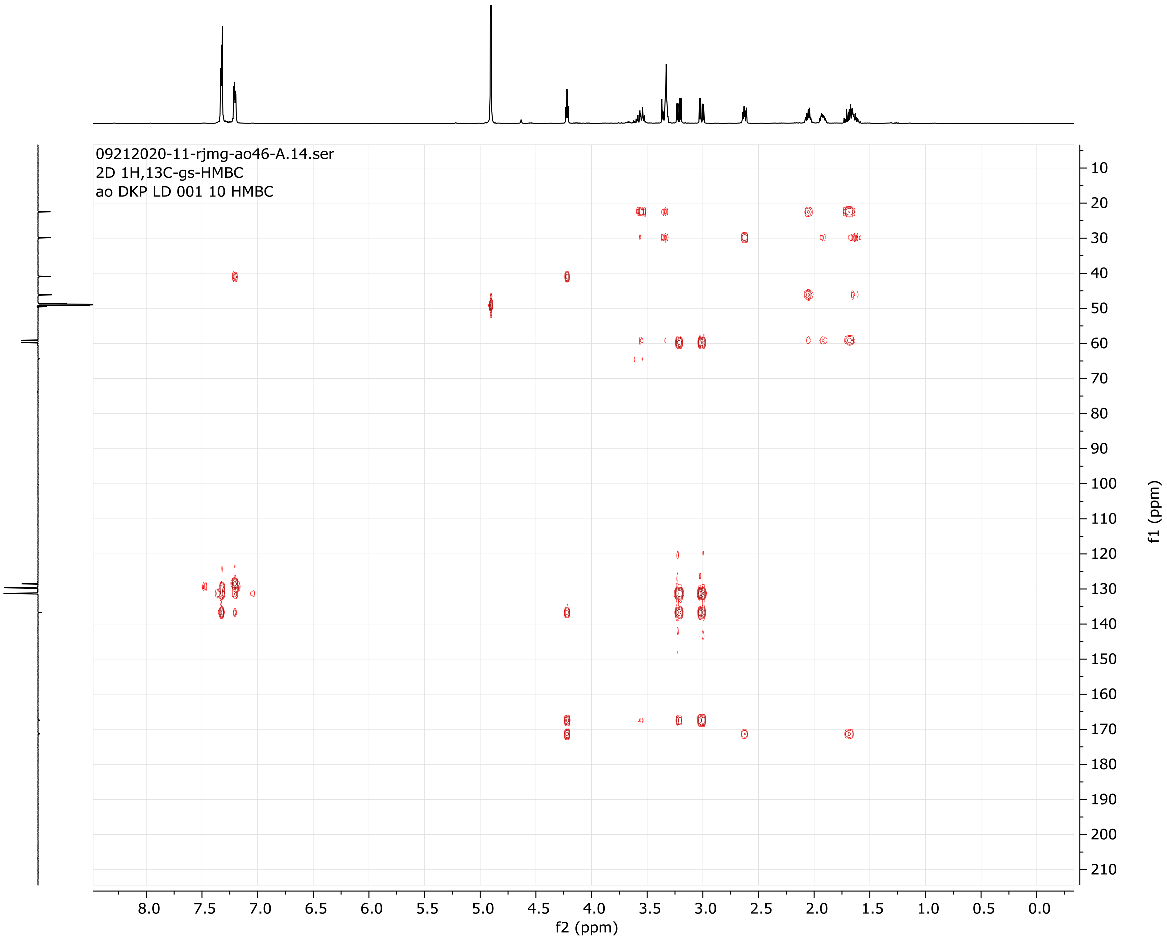

HMBC NMR of cyclo-(D-Phe-L-Pro)
